# Environmental Durability of an Optical Fiber Cable Intended for Distributed Strain Measurements in Concrete Structures

**DOI:** 10.3390/s22010141

**Published:** 2021-12-26

**Authors:** Ismail Alj, Marc Quiertant, Aghiad Khadour, Quentin Grando, Karim Benzarti

**Affiliations:** 1 Matériaux et Structures (MAST) Department, Expérimentation et Modélisation pour le Génie Civil et Urbain (EMGCU), University Gustave Eiffel—Institut Français des Sciences et Technologies des Transports, de l’Aménagement et des Réseaux (IFSTTAR), F-77447 Marne-la-Vallée, France; marc.quiertant@univ-eiffel.fr; 2 Composants et Systèmes (COSYS) Department, Laboratoire Instrumentation, Simulation et Informatique Scientifique (LISIS), University Gustave Eiffel—Institut Français des Sciences et Technologies des Transports, de l’Aménagement et des Réseaux (IFSTTAR), F-77447 Marne-la-Vallée, France; aghiad.khadour@univ-eiffel.fr; 3 Institut de Radioprotection et de Sûreté Nucléaire (IRSN), CEDEX, 13115 Saint-Paul-Lez-Durance, France; quentin.grando@irsn.fr; 4 Lab Navier, University Gustave Eiffel, Ecole Nationale des Ponts et Chaussées (ENPC), Centre National de la Recherche Scientifique (CNRS), F-77447 Marne la Vallée, France

**Keywords:** distributed optical fiber sensors, strain measurement, durability, accelerated and natural ageing conditions, degradation kinetics, pull-out tests, strain transfer process

## Abstract

The present study investigates the environmental durability of a distributed optical fiber sensing (DOFS) cable on the market, commonly used for distributed strain measurements in reinforced concrete structures. An extensive experimental program was conducted on different types of specimens (including samples of bare DOFS cable and plain concrete specimens instrumented with this DOFS cable) that were exposed to accelerated and natural ageing (NA) conditions for different periods of up to 18 months. The instrumentation of both concrete specimens consisted of DOFS cables embedded at the center of the specimens and bonded at the concrete surface, as these two configurations are commonly deployed in the field. In these configurations, the alkalinity of the surrounding cement medium and the outdoor conditions are the main factors potentially affecting the characteristics of the DOFS component materials and the integrity of the various interfaces, and hence impacting the strain transfer process between the host structure and the core optical fiber (OF). Therefore, immersion in an alkaline solution at an elevated temperature or freeze/thaw (F/T) and immersion/drying (I/D) cycles were chosen as accelerated ageing conditions, depending on the considered configuration. Mechanical characterizations by tensile and pull-out tests were then carried out on the exposed specimens to assess the evolution of the mechanical properties of individual component materials as well as the evolution of bond properties at various interfaces (internal interfaces of the DOFS cable, and interface between the cable and the host structure) during ageing. Complementary physico-chemical characterizations were also performed to better understand the underlying degradation processes. The experimental results highlight that immersion in the alkaline solution induced a significant and rapid decrease in the bond properties at internal interfaces of the DOFS cable and at the cable/concrete interface (in the case of the embedded cable configuration), which was assigned to chemical degradation at the surface of the cable coating in contact with the solution (hydrolysis and thermal degradation of the EVA copolymer component). Meanwhile, F/T and I/D cycles showed more limited effects on the mechanical properties of the component materials and interfaces in the case of the bonded cable configuration. A comparison with the same specimens exposed to outdoor NA suggested that the chosen accelerated ageing conditions may not be totally representative of actual service conditions, but provided indications for improving the ageing protocols in future research. In the last part, an analysis of the distributed strain profiles collected during pull-out tests on instrumented concrete specimens clearly illustrated the consequences of ageing processes on the strain response of the DOFS cable.

## 1. Introduction

With progress in the field of construction materials and engineering techniques, new civil structures are expected to remain in service for many decades [1]. Together with a robust design and the use of high-performance materials, structural health monitoring (SHM) has proven to be an effective approach for ensuring the safety/reliability of concrete infrastructures over the long term [2,3,4,5,6,7]. Among existing SHM techniques, distributed optical fiber sensor (DOFS) systems [8,9,10,11,12,13,14] have gained interest for strain measurement and crack monitoring applications, as they enable one to collect data continuously using a sensor with a sub-millimeter spatial resolution [7,15,16] over long distances [17,18] and with low intrusiveness compared to conventional sensors [11,19]. In practice, DOFS can either be bonded to the surface of existing structures [20,21] or embedded within reinforced concrete (RC) elements during construction (most often attached to the steel rebars) [19,22]. Depending on the configuration, DOFS are thus either exposed to weather conditions [23,24] or subjected to the alkaline environment of the cement matrix [6,19,25], which both may affect the physical/mechanical properties of their component materials over time. Under such complex environments, knowledge on the long-term durability/reliability of DOFS remains very limited. This issue is indeed rarely addressed in the literature, although a few studies have reported that the component materials of optical fiber (OF) sensors, such as primary coatings or external polymer claddings, can undergo chemical degradation under thermo-oxidative/hydrolytic conditions [26], or microstructural changes under the effect of thermal treatments (physical ageing process) [23]. In addition, when manufacturers generally claim that their products have good durability, they rarely provide supporting experimental evidence or quantified durability performance in their technical documentation, hence raising questions and doubts among end-users.

To ensure effective mechanical and chemical protection of the sensors, thick DOFS cables are generally used instead of bare OF protected by their primary coating only. DOFS cables contain one or several standard OFs surrounded by various coating layers and mechanical reinforcements [27]. These additional layers, although necessary for protective functions, are prone to shear deformation and strongly influence the strain transfer process between the host concrete structure and the core OF (the sensing part of the cable), which can make the interpretation of raw data hazardous. Therefore, the determination of a mechanical transfer function (MTF) is generally required to account for this shear lag effect and produce accurate quantitative measurements [28,29,30]. As much as physical/chemical ageing processes affect the mechanical properties of the DOFS protective layers and the integrity of interfaces over time, they also alter the shear lag effect and decrease the reliability of strain data derived using the MTF of the reference (unaged) system. For all these reasons, it is of paramount importance to assess any changes in the mechanical properties of DOFS component materials under service conditions, as well as their possible influence on the strain transfer process. The step further would be to describe the evolution of the MTF over the entire service life of the instrumentation (such an evolution is specific to each DOFS cable in a given service environment), as this knowledge would ensure the reliability of strain measurements in the long-term. However, this objective is outside of the scope of the present paper.

The main purpose of this work is indeed to evaluate the degradation behavior of a particular DOFS cable on the market when exposed to accelerated ageing conditions that are considered representative of actual service environments. To this end, a comprehensive durability test program was designed, including different types of specimens:

- Samples of bare DOFS cable were used to investigate exposure effects on the mechanical properties/the microstructure of the component materials and internal interfaces of the selected cable,
- Concrete specimens containing the DOFS cable were subjected to pull-out tests, with a view to monitoring possible changes in the bond properties between the sensor and the host concrete structure during the ageing process. Two specimen geometries were considered to account for actual configurations used in the field, namely concrete cylinders with DOFS cables embedded along the central axis (denoted embedded configuration) and concrete prisms with DOFS cables bonded at the surface of each face (denoted bonded configuration),
- Additional samples (concrete cylinder and samples of bulk polymer adhesive) were also considered to assess possible changes in the properties of the host concrete or the adhesive (used in the bonded configuration) over the course of the ageing process.

In order to simulate the ageing of an embedded DOFS instrumentation under laboratory conditions, the dedicated specimens were exposed to a highly alkaline solution (pH~13.5) representative of the interstitial concrete pore solution for various periods of up to 18 months. This exposure was carried out at room temperature and also at higher temperatures to accelerate the degradation processes. On the other hand, to artificially reproduce the ageing of a bonded DOFS instrumentation exposed to weather conditions, corresponding specimens were subjected to temperature/moisture cycles. In addition, series of specimens were also exposed to outdoor conditions in the Paris area (France), in order to compare the effects of accelerated ageing conditions to those induced by a natural ageing (NA) process. The effect of sustained load (creep) on the mechanical characteristics of the DOFS component materials and internal interfaces was also briefly addressed in the framework of this study.

The first part of the paper presents the overall program of this durability study, providing further details on the characteristics of the selected DOFS cable, the preparation of the test specimens, the ageing protocols, and the experimental techniques used for mechanical, physical and chemical characterizations of specimens in the initial/aged states. 

In a second part, the changes in mechanical properties that were observed during ageing for the cable’s protective coating and for the various interfaces (internal interfaces, as well as cable/concrete or cable/adhesive interfaces) are reported and discussed in the light of the chemical degradation/microstructural evolution of the component materials revealed by physico-chemical characterizations. 

Finally, some distributed strain profiles collected with a Rayleigh interrogator during pull-out tests on the reference and aged specimens are also presented in order to illustrate the influence of ageing-induced degradation on the response of the DOFS sensor. 

It is worth noting that our results are specific to the particular DOFS cable considered in this study, but the overall methodology proposed for investigating the environmental durability can be reproduced for any other cable on the market.

## 2. Experimental Program

### 2.1. Characteristics of the Selected DOFS Cable

**Figure 1:**
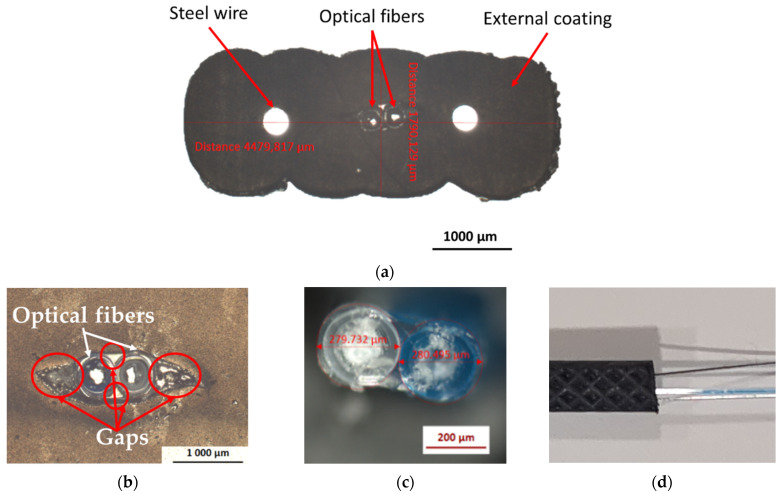
Optical microscope observations of (**a**) the cross-section of the DOFS cable, (**b**) the cross-section of the central part of the cable, and (**c**) the cross-section of the two central OFs; (**d**) picture showing the external surface relief of the cable, as well as an uncoated segment.

The DOFS cable considered in this study was the FutureNeuro™ FN-SILL-3 cable manufactured by Neubrex Co., Ltd. (Kobe, Japan) [31]. This cable is intended for strain sensing applications and can be installed both at the surface of and embedded within the host structure. It is specifically designed with an embossed surface geometry to promote adhesion with concrete (Figure 1a,d). As illustrated in the pictures of Figure 1, this cable is composed of:

- Two single-mode OFs with an acrylate primary coating;
- Two steel wires (usually referred as strength members) with a diameter of 0.3 mm, which ensures the mechanical reinforcement of the cable;
- An external sheath (≈4.5 mm × 1.7 mm) made of soft polyolefin elastomer, which provides chemical and mechanical protection of the cable.

According to the supplier’s data sheet [31], the cable offers high thermal resistance and can be used in a temperature range from −20 to 80 °C.

Optical microscope observations of the “as received” cable show the presence of few gaps at the interface between the OF primary coatings and the external elastomer coating (Figure 1b), probably formed during the manufacturing process. It can be expected that these gaps will influence the strain transfer from the host structure to the core silica fiber of the DOFS cable (i.e., the sensing element). Meanwhile, the interfaces between the steel wires and the external coating are sound, and do not present any visible defects.

### 2.2. Description and Preparation of the Test Specimens

**Figure 2:**
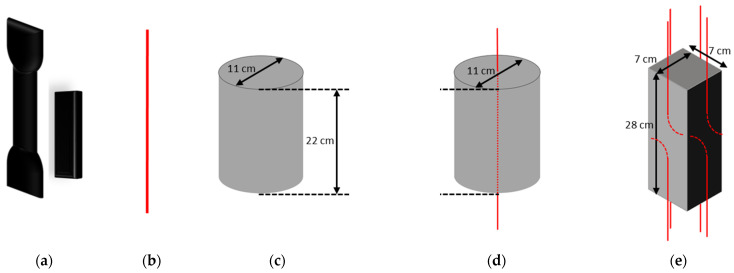
Schematic descriptions of the different specimens: (**a**) samples of bulk adhesive (**b**) samples of DOFS cable, (**c**) concrete cylinders, (**d**) concrete cylinders with embedded DOFS cables and (**e**) concrete prisms with DOFS cables bonded on the 4 lateral faces.

Different types of test specimens were designed to cover the needs of this durability study, and in particular for performing mechanical characterization in the initial state and after different ageing periods of: the adhesive used for bonding the DOFS cable to the concrete surface (Figure 2a), the component materials of the cable itself (Figure 2b), the concrete material (Figure 2c), the cable/concrete interface in the case of an embedded cable configuration (Figure 2d) and the cable/adhesive or adhesive/concrete interfaces in the case of a bonded cable configuration (Figure 2e). Related sample geometries are depicted in Figure 2.

The samples of DOFS cable (Figure 2b) were intended for conducting tensile tests on the component materials (external coating and reinforcing steel wires), as well as pull-out tests on internal interfaces (i.e., external coating/OF and external coating/steel reinforcement interfaces). They were also used for the physico-chemical characterizations. The DOFS cable was stripped in order to prepare external coating and steel wire specimens for tensile tests, or partially stripped to prepare specimens intended for external coating/OF and external coating/steel wire pull-out tests.

Regarding the polymer adhesive, we selected a bi-component epoxy system X120 commercialized by HBM company (Darmstadt, Germany), which is commonly used for bonding optical and electrical gauges in various industrial applications. Dumbbell samples of the bulk adhesive (Figure 2a left) complying with EN-ISO 527-2 standard [32] were molded for mechanical tensile tests, whereas small prismatic samples of size 50 × 12 × 2 mm^3^ (Figure 2a right) were molded for physio-chemical characterizations. Polymerization of the samples was achieved in the laboratory at 20 °C and ~50% relative humidity (RH).

Concrete specimens were prepared with a CEM 1 Portland cement content of 375 kg/m^3^ and a water to cement ratio (*W*/*C*) of 0.48. All specimens were cast from the same batch of concrete, including:

- Cylinders with a diameter of 11 cm and a height of 22 cm were used in compression tests (Figure 2c). After demolding, these cylinders were cured in water at room temperature for 3 months to advance the hydration process of concrete. Exposure tests started at the end of this curing period (=time T0 of the durability program),
- Instrumented concrete cylinders (diameter 11 cm, height 22 cm) containing a DOFS cable embedded along the central axis (Figure 2d) were subjected to pull-out tests for determining the bond characteristics of the cable/concrete interface in the embedded cable configuration. Here, again, a 3-month curing period in water at room temperature was applied prior to initial characterizations on control specimens and the conditioning of exposed specimens in their ageing environments (=time T0),
- Instrumented concrete prisms (dimensions 7 × 7 × 28 cm^3^) bearing DOFS cables bonded on each of the four lateral faces (Figure 2e) were used for pull-out tests to evaluate the bond properties between the bonded cable and the host structure (i.e., at the cable/adhesive or at the adhesive/concrete interfaces, depending on the pull-out failure mode). The cables were bonded a few days after the demolding of the concrete prisms (see the bonding procedure in the next paragraph), and the instrumented prisms were again cured for 3 months in water at an ambient temperature until time T0. It is worth noting that the samples of bulk X120 adhesive introduced previously were also subjected to an immersion period of 3 months, in order have the same history at time T0 compared to the adhesive joints of instrumented concrete prisms.

**Figure 3:**
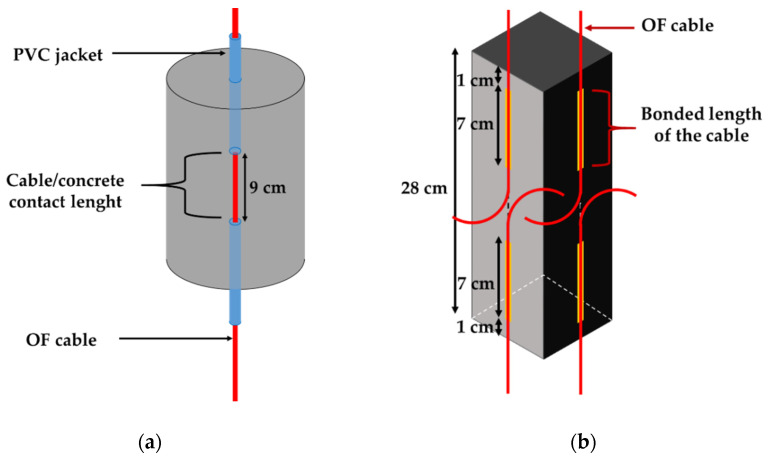
Schematic descriptions of (**a**) a concrete cylinder instrumented with a DOFS cable embedded over a length of 9 cm; (**b**) a concrete prism equipped with two DOFS cables per face, bonded over lengths of 7 cm.

Since instrumented concrete specimens were intended to assess possible changes in bond properties during ageing, it was necessary to select adequate contact lengths (i.e., embedment length or bonded length) that promote interfacial bond failure instead of tensile failure of the DOFS cable itself. Preliminary tests were thus carried-out to optimize these contact lengths, and values of 9 and 7 cm were retained for the embedded and bonded configurations, respectively. A PVC jacket was used as a “bond breaker” to set the embedment length in the cylinder specimens (see Figure 3a). Regarding the bonded configuration, two DOFS cables were bonded on each face of the prismatic specimens (see Figure 3b). The bonding procedure is detailed in [11] and requires engraving grooves at the concrete surface (approximate depth and width of 2 mm) prior to sealing the cables in the grooves using the X120 epoxy adhesive, in order to ensure better adhesion. For each face, the two cables were inserted in a same unique groove, but they were sealed at the opposite extremities of the prism over a contact length of 7 cm and 1 cm away from the edges. One prismatic specimen was thus equipped with a total of 8 bonded cables, allowing us to perform 8 separate pull-out tests.

### 2.3. Characterization Methods

#### 2.3.1. Mechanical Characterizations of the Test Specimens

**Figure 4:**
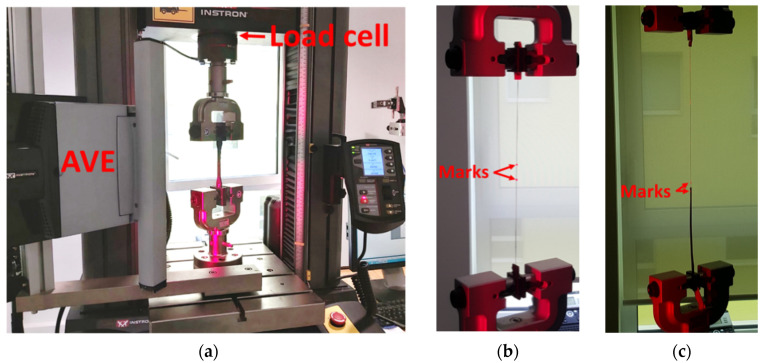
Tensile tests on (**a**) a dumbbell sample of X120 adhesive and on (**b**) a steel wire reinforcement (showing the two marks used for AVE monitoring); (**c**) typical pull-out test setup used to determine the bond properties at the external coating/steel wire interface (showing the two marks used to measure differential displacement between adherents).

Tensile tests were performed on the dumbbell samples of bulk X120 adhesive, in the initial state at T0 and after different ageing periods. Tests were made according to EN-ISO 527-2 standard [32] using a universal testing machine (UTM), model 5969 from Instron company (Norwood, MA, USA), equipped with a load cell of capacity 2 kN, and a non-contact Advanced Video Extensometer (AVE) as shown in Figure 4a. This AVE device enables tracking the relative displacements of two marks plotted at the sample surface and provides strain values during the test. The resolution on the force measurements was 2 N, and the resolution of AVE measurements was 0.5 μm, with an accuracy of 0.5% of reading. Results were averaged for three repeated tests.

**Figure 5:**
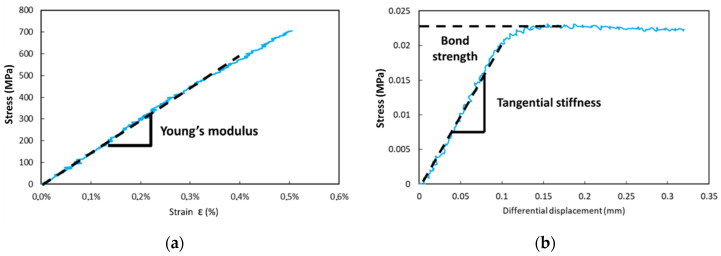
(**a**) Typical tensile curve for a steel wire reinforcement, and (**b**) typical pull-out curve for the external coating/OF interface in the initial state (unaged sample).

Some component materials of the DOFS cable (i.e., the external coating and the steel reinforcements) were also tested in tension (Figure 4b) before ageing at T0 and after various exposure periods, in order to assess possible changes in their Young’s moduli. This implied stripping the cable to separate the individual components before testing. A typical tensile curve obtained for steel wire reinforcements is displayed in Figure 5a. In addition, the mechanical properties of several internal interfaces of the DOFS cable (external coating/OF interface and external coating/steel reinforcement interface) were characterized by pull-out tests using the same Instron 5969 UTM. In this case, the AVE measured the differential displacement between marks plotted on each adherent material (Figure 4c), whilst the interface was subjected to shear loading at a controlled displacement rate of 1 mm/min. A typical pull-out curve obtained for the external coating/OF interface is illustrated in Figure 5b. From the slope of the initial linear part of the curve, one can determine a tangential stiffness of the considered interface (in MPa/mm), while the maximum stress value provides the bond strength (in MPa). Results were averaged over a series of 3 tests. It should be mentioned that the preparation of specimens and their clamping in the UTM grips was not easy, and might sometimes have induced slight damage to the samples. Therefore, the results of these pull-out tests should not be considered as absolute quantitative data, but rather as a way to highlight variation trends during ageing.

Compression tests were conducted on the concrete cylinders according to the NF EN 12390 standard [33], 28 days after casting and after various ageing periods. They were performed with a RK-MFL UTM with a capacity of 5000 kN, and this allowed us to determine the concrete compressive strength, Young’s modulus and Poisson’s ratio. Mean values resulted from three repeated tests.

**Figure 6:**
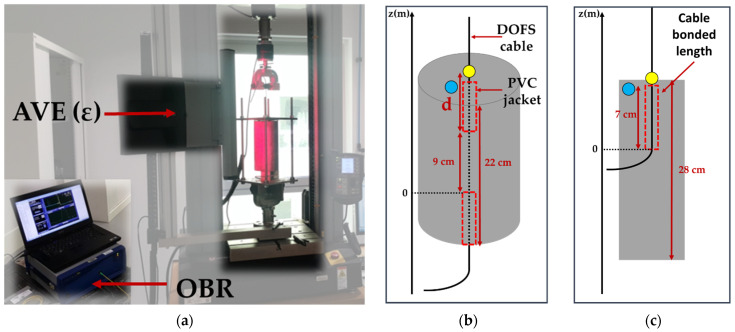
Characterization of the bond properties between the DOFS cable and the host concrete specimens: (**a**) pull-out tests setup, and location of the marks tracked by the AVE for (**b**) the embedded cable and (**c**) the bonded cable configurations.

Pull-out tests were also carried on the instrumented concrete specimens in order to determine the bond properties between the DOFS cable and the concrete, in the initial state (at T0) and after different ageing periods as well. Both concrete cylinders with embedded DOFS cables and concrete prisms with bonded DOFS cables were tested using the same Instron 5969 UTM equipped with the AVE system. A specific steel frame was designed to hold the concrete specimens during pull-out tests, with holes drilled on the upper plate to let the DOFS cable pass through. The frame was attached to the fixed lower grip of the UTM, while the cable was clamped in the mobile upper grip, as shown in Figure 6a. All pull-out tests were performed at a displacement rate of 1 mm/min, and the displacement was stopped for 1 min every 20 N load step. During these periods, one of the two OFs of the cable was interrogated using a Rayleigh Optical Backscatter Reflectometer (OBR), model 4600 from LUNA Innovations (Roanoke, VA, USA). Such measurement provides a continuous strain profile along the OF. In addition, for AVE tracking, two marks are placed on the test specimen: one mark is fixed at the top face of the concrete specimen (see the blue mark in Figure 6), and a second is placed on the cable part subjected to pull-out load (yellow mark in Figure 6). The differential displacement *u_d_* between the two marks is continuously monitored using the AVE camera. Regarding the embedded cable configuration (Figure 6b), *u_d_* results from the contribution of two parts: the slip at the cable/concrete interface, denoted as *u_i_*, and the displacement *u_t_* related to the tensile strain of the cable length *d* between the yellow mark on the cable and the location where the cable exits from concrete. The interrogation of the cable using the OBR device enables the calculation of the cable stiffness, the deduction of *u_t_* and finally the extraction of the bond slip *u_i_ = u_d_ − u_t_*, whose evolution with load describes the cable/concrete interface behavior. For the bonded cable configuration, the situation is simpler, as *u_d_* is actually equal to *u_i_*, as illustrated in (Figure 6c). These pull-out experiments enabled us to plot bond stress/slip curves which have a typical aspect very similar to that of Figure 5b (note that the bond stress is calculated as the ratio of the applied load to the embedded/bonded surface area, in N/m^2^). The initial slope of the curve gave then access to the tangential stiffness of the interface (in MPa/mm), while the maximum stress reached just before the sliding of the cable corresponded to the bond strength (in MPa). Here, again, all values were averaged over 3 repeated tests.

#### 2.3.2. Physical and Chemical Characterizations of the Specimens

In addition to the previously described mechanical tests, complementary investigations were carried out to reveal possible ageing-induced changes in several physical and chemical characteristics of the specimens and their component materials:

- The transverse dimensions of the DOFS cable (thickness and width) were monitored during ageing. Measurements were made on a periodical basis at 4 different cross-sections of a single cable sample, which allowed us to calculate an average value of the thickness and width, as well as their standard deviations;
- Gravimetric measurements were also carried out to assess the water sorption behavior of the bulk X120 adhesive. Small prismatic samples of approximate weight 1.5 g were immersed in water at room temperature (20 °C), and were then periodically removed for weighing with a balance with a resolution of 1 mg. Before measurements, the surface of samples was dried with a tissue. Mass gain was monitored on 8 samples to provide mean values and standard deviations.
- Surface observations of the external coating of DOFS cables performed made before and during ageing, using an optical microscope AxioScope A1 from Carl Zeiss AG (Oberkochen, Germany), in order to detect possible surface damage;
- The external coating of the DOFS cable and the samples of bulk X120 adhesive were also analyzed using Fourier-transform infrared spectroscopy (FTIR) and differential scanning calorimetry (DSC) in order to identify their composition and reveal possible alterations to the chemical structure or microstructural changes of the polymer network during ageing. FTIR analyses were carried out using a Nicolet IS 50 IRTF Spectrometer from Thermo Fisher Scientific (Waltham, MA, USA), equipped with a diamond Attenuated Total Reflectance (ATR) device. Spectra were recorded in a wavelength range from 4000 to 400 cm^−1^, with a resolution of 4 cm^−1^ and an accumulation of 32 scans. DSC analyses were performed using a Discovery DSC 250 calorimeter from TA Instruments (New Castle, DE, USA). For the cable’s external coating, DSC tests were carried out on 15–20 mg samples, to which a temperature ramp from −70 to 230 °C was applied at a heating rate of 20 °C/min. Regarding the X120 adhesive, analyses were carried out in MDSC mode (DSC with temperature modulation), which helped to separate the glass transition from other thermal events. Samples with a weight of 10 mg were subjected to a linear temperature ramp from −10 to 130 °C at a heating rate of 2 °C/min, with a superimposed temperature modulation of amplitude 1.5 °C and period 60 s. The glass transition of the polymer was easily detected as an endothermic jump on the reversing heat flow component, and the glass transition temperature (*Tg*) was then identified at the inflexion point of the curve.

### 2.4. Ageing Protocols

To investigate the durability of the DOFS cable embedded in concrete or bonded on its surface, the different types of specimens and samples presented in Section 2.2 were subjected to accelerated ageing in different environments, according to the following protocols. Some specimens were also exposed to NA in outdoor conditions for the sake of comparison. The influence of creep under sustained load (Cr) was also investigated.

#### 2.4.1. Ageing Protocol for Embedded DOFS Cables

To investigate the effect of alkalinity from the cement medium on the embedded DOFS cables, instrumented cylinders were immersed in an alkaline solution to undergo chemical attack. Considering that temperature influences the degradation kinetics of the cable, several temperatures of the alkaline solution were used to modulate the acceleration rate: 20, 40 and 60 °C. In addition, to evaluate the effects of ageing on individual components of the system, plain concrete cylinders (without cable) and samples of bare DOFS cable were also exposed to the same conditions. 

**Figure 7:**
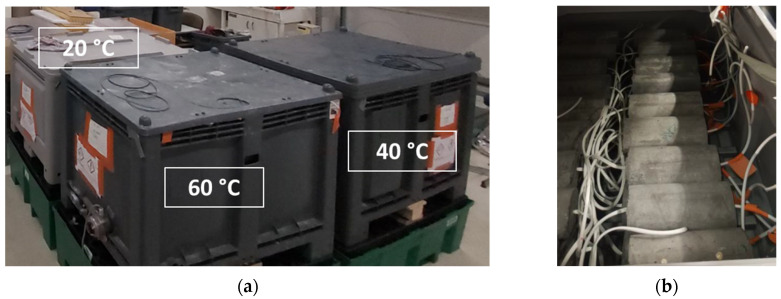
Conditioning in the alkaline ageing environment: (**a**) thermo-regulated tanks filled with the alkaline solution, and (**b**) instrumented concrete cylinders immersed in the solution.

The alkaline solution was composed of sodium hydroxide (NaOH) and potassium hydroxide (KOH) at the same concentrations as those adopted in previous durability studies [19,34] and specified in Table 1. The pH of this solution is around 13.5, and is considered as representative of the alkalinity of concrete at an early age. An overall volume of 0.8 m^3^ of this solution was prepared and distributed in 3 thermo-regulated tanks (one tank per considered temperature), as shown in Figure 7a. 

The accelerated ageing program lasted 18 months and included sample characterizations every 3 months. The idea was to assess the possible degradations of the DOFS instrumentation on a regular basis while keeping a reasonable work plan. 

In the following, time T0 corresponds both to the date on which the specimen’s exposure started, and to the date of the initial characterizations of control specimens.

#### 2.4.2. Accelerated Ageing Protocol for Bonded DOFS Cables

In the same way, the prismatic concrete specimens equipped with bonded DOFS cables were subjected to accelerated ageing conditions representative of their actual in-service environment. Contrary to the case of embedded cables, bonded cables are sealed in grooves at the surface of concrete with thin layers of adhesive, which prevents them from direct contact with the alkaline concrete substrate on the one hand, but offers limited protection against external weather conditions on the other. In the long term, such an exposure to weather conditions may: (i) affect the response of the instrumentation itself, by altering/damaging materials (component materials of the DOFS cable or adhesive layer) or interfaces (i.e., cable/adhesive or adhesive/concrete interfaces), and also (ii) induce cracking at the surface of concrete, hence affecting the integrity of the adhesive/concrete interface. 

Considering that temperature and humidity variations are the main factors responsible for possible degradations in this configuration, accelerated ageing protocols should thus simulate in an accelerated way the daily temperature cycles as well as the alternation of rainy/dry periods at certain periods of the year. In this context, it was decided to consider freeze/thaw (F/T) and immersion/drying (I/D) cycles, which can be representative of severe weather conditions. 

**Figure 8:**
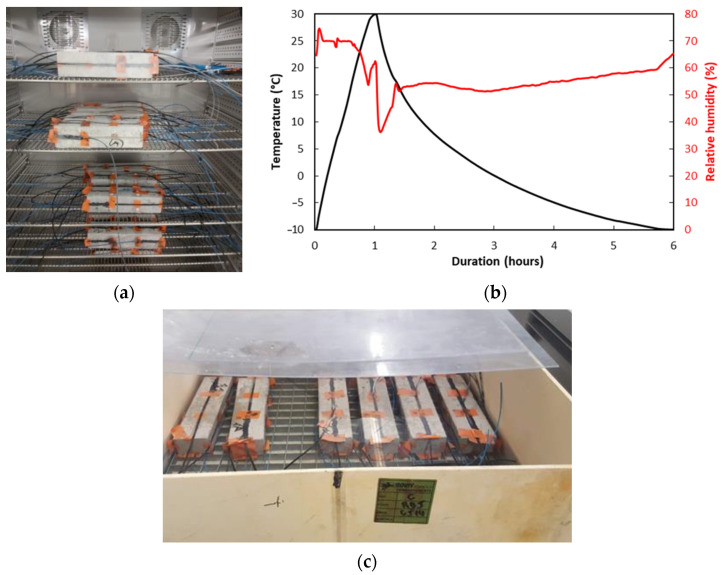
Application of cyclic conditions: (**a**) specimens subjected to F/T cycling in a climatic chamber and (**b**) Temperature/RH variations recorded in the chamber over a F/T cycle. (**c**) Specimens exposed to I/D cycling in a tank.

F/T cycling was conducted in a climatic chamber (Figure 8a) over a total period of 18 months. These cycles aimed at simulating fictitious daily temperature alternation (with a deliberately high amplitude from +30 to −10 °C, based on extreme summer/winter temperatures encountered in the Paris area, to increase the test severity), in an accelerated manner (cycles of 6 h, corresponding to an acceleration rate of 4 compared to daily alternation). Typical temperature/humidity variations recorded during a F/T cycle are displayed in Figure 8b. One can note that a duration of 5 h was necessary to achieve cooling from 30 to −10 °C, as the chamber was not able to maintain a constant cooling rate in the whole domain. In addition, the RH level was only controlled in the heating phase (RH~70%), but could not be regulated during the cooling phase.

The protocol of I/D cycles consisted of alternating a one-week immersion period in a water tank at room temperature with a one-week drying period in ambient air (Figure 8c). Water was renewed in the tank at each cycle, to favor the leaching of concrete. An overall exposure period of 18 months was initially scheduled (as for the other accelerated conditions), but it was finally reduced to 16 months due to the COVID-19 pandemic. Here, again, T0 denotes the starting date of exposure.

#### 2.4.3. Natural Ageing in Outdoor Conditions (NA)

**Figure 9:**
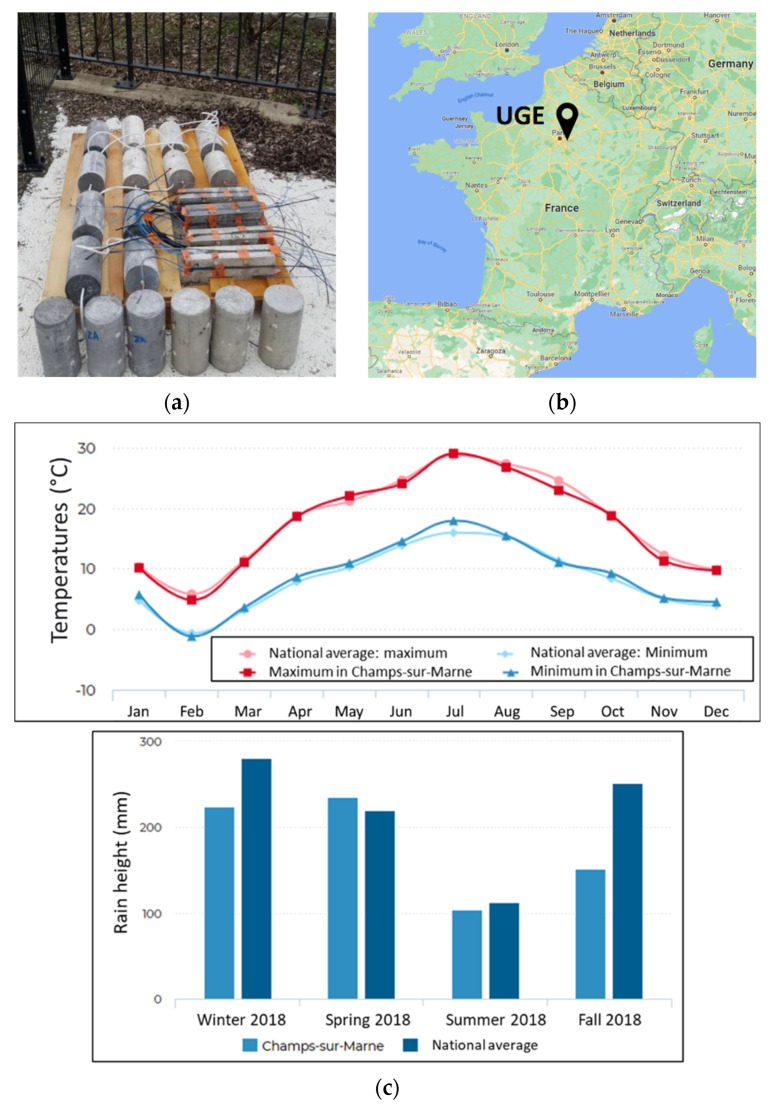
(**a**) Storage of specimens on the natural ageing site, (**b**) geographical location of the site of the Université Gustave Eiffel (UGE) in Champs-sur-Marne, France [35] and (**c**) local weather data [36].

The previous accelerated ageing environments were intended to simulate the long-term behaviour of the embedded/bonded DOFS cable instrumentation in service conditions. However, the relevance of simulated conditions is often questionable, and the correspondence with actual service conditions in terms of acceleration rate remains a critical issue. To confirm the relevance of accelerated tests and verify that degradation mechanisms are similar to those induced by natural outdoor exposure, series of specimens of the various types were stored on a NA site located in Champs-sur-Marne (Paris area, France) over a period of 16 months. Figure 9 depicts the location of the site, as well as the average monthly temperatures and seasonal rainfall.

#### 2.4.4. Creep Test Setup for DOFS Cables

**Figure 10:**
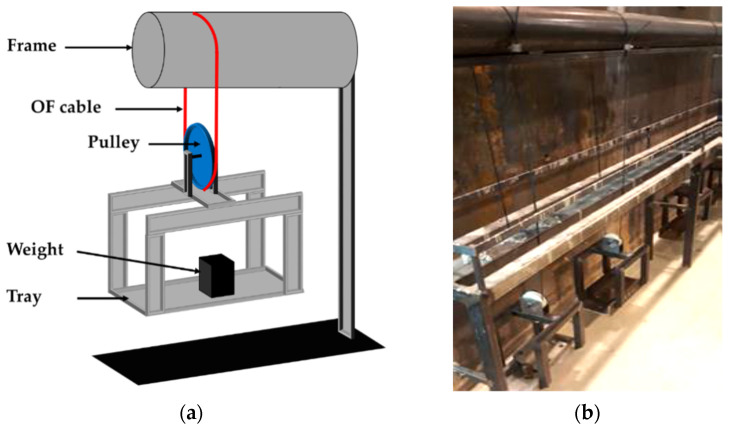
Creep test setup: (**a**) Schematic description and (**b**) picture of the setup.

In addition to the previous environmental factors, civil infrastructures are also subjected to sustained load, mostly due their own weight (dead load). Consequently, both embedded and bonded DOFS cable instrumentations may undergo creep loading under service conditions. It was therefore decided to include a specific creep test (denoted Cr) in this experimental program, in order to study possible creep-induced changes in the mechanical and geometrical properties of the DOFS cable. This test consisted of applying a constant tensile load to the DOFS cable for a total period of 15 months. To this purpose, three metallic setups were specifically designed and manufactured in the laboratory, as illustrated in Figure 10. Each setup was equipped with a tray to hold the weights used for the application of sustained load. This tray is connected to a pulley that ensures load balancing in the DOFS cable forming a closed loop. Each tray holds a weight of 7.5 kg, all included.

#### 2.4.5. Summary of the Experimental Program

To facilitate the analysis of the next sections, the experimental program of this durability study is summarized in Table 2. The different characterizations performed at each session are recalled, with the number of repeated tests is given in brackets. The term “other analyses” refers to the complementary physico-chemical characterizations, OM corresponds to the observations by optical microscopy, and D to the determination of the cable dimensions.

One should keep in mind that the different accelerated or NA tests did not start at the same calendar dates during the project. Nevertheless, for all series, T0 corresponds to the moment of the initial characterizations on control specimens, on which the exposed specimens were also placed in their ageing environments. 

## 3. Experimental Results

In this section, results of the characterization tests performed on the different types of specimens before ageing (at T0) and after different exposure times (test sessions T1 to T6) are presented. These experimental results reveal possible changes in the specimen’s mechanical properties under the effect of the various ageing environments, and provide information regarding the degradation processes involved at the microstructural scale.

### 3.1. Initial Characterizations on Control Specimens

#### 3.1.1. Initial Characterization of Concrete

The concrete cylinders were cured in water and tested in compression 28 days after casting as specified in the standard method (cf. Section 2.3.1). The results of this initial characterization on control specimens are reported in Table 3. The results obtained for aged concrete cylinders are presented at a later stage in this article.

#### 3.1.2. Mechanical Characterization of the DOFS Cable at T0

Initial characterizations by tensile and pull-out tests were performed at T0 to determine the tensile properties of several material components of the DOFS cable (external coating and steel wire reinforcement), as well as the bond behavior at internal interfaces (external coating/steel reinforcement and external coating/OF, see Section 2.3.1). Table 4 presents the experimental results, where the tangential stiffness corresponds to the slope of the bond stress/slip curve at the considered interface. It should be remembered that the bond stress is calculated by dividing the applied load by the contact surface area between the layers subjected to shear stress during the pull-out test. 

As previously underlined, these experimental data should be considered with caution, as slight damage of the cable components or interfaces may have occurred during sample preparation, especially when stripping the cable to separate the internal components. Such hazardous damage may also explain the large dispersion observed for some characteristics. Nevertheless, considering that the same stripping protocol is applied during the whole experimental campaign, it is possible to compare these data to those obtained on aged samples in order to draw general trends. This comparison is made in a later section of this paper.

#### 3.1.3. Characterization of the Bulk Adhesive at T0

Before starting ageing tests on concrete prisms instrumented with externally bonded DOFS cables, these specimens were stored in water for 3 months to achieve the curing of concrete (cf. Section 2.2). In addition, the cyclic ageing protocols (I/D cycles or F/T cycles) involved direct exposure of the X120 adhesive with the surrounding wet environment (water or moist air). For these reasons, it was important to evaluate the effects of water absorption on the mechanical properties of this polymer adhesive. 

**Figure 11:**
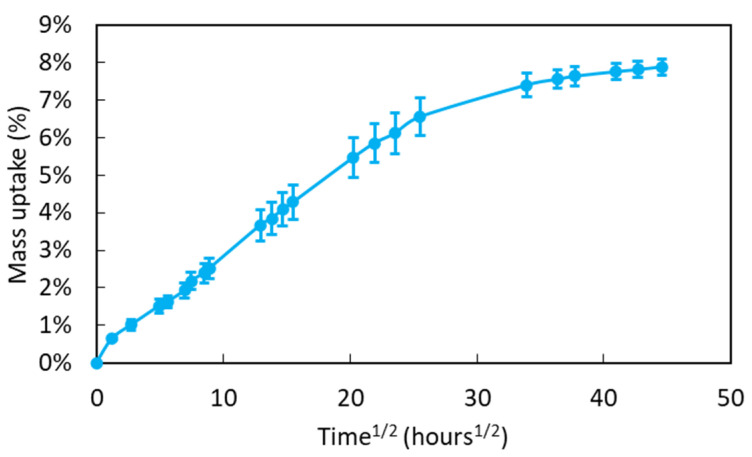
Absorption curve of the bulk adhesive samples immersed in water at 20 °C.

Figure 11 displays the mass uptake versus the square root of immersion time for samples of bulk adhesive immersed in water at room temperature. The shape of this sorption curve exhibits an initial linear part followed by a progressive decrease in the sorption kinetics, suggesting that this is controlled by a Fickian diffusion process [37]. After 80 days of exposure, the mass uptake is close to 7.8%, and the saturation plateau is not totally reached. From the apparent trend, one can expect an equilibrium water content in the range of 8–8.5%.

In addition, tensile tests were performed on two series of bulk adhesive samples. The first series was conditioned in water for 3 months prior to these tests (the same history at time T0 as that of concrete prisms instrumented with bonded DOFS cable). The second series was kept in ambient air for the same period. Table 5 reports the tensile properties determined on the two series (mean values and standard deviations on three specimens).

These test results show that the preliminary immersion period in water had a significant effect on the mechanical properties of the bulk adhesive, as both the elastic modulus and tensile strength are much lower compared to those of the series conditioned in air (about −34% and −40%, respectively), which is consistent with the literature. According to [38,39], the absorption of water by a cross-linked epoxy network induces an overall increase in molecular mobility and a decrease in the glass transition temperature (*Tg*). This plasticization phenomenon of the network by water molecules generally entails a significant decrease in the mechanical properties of the polymer (Young’s modulus, tensile strength, hardness index), as well as an increase in ductility [39]. 

Finally, one should keep in mind that, at the beginning of the durability test program (at time T0), the X120 adhesive layer present on concrete prisms instrumented with bonded DOFS cables will have lower ultimate properties and higher ductility compared to the same polymer stored under ambient laboratory conditions, due to the preceding curing schedule of the prisms in water.

#### 3.1.4. Mechanical Characterization of the DOFS Cable/Concrete and DOFS Cable/Adhesive Interfaces at T0

Initial pull-out characterizations were carried out at time T0 on control concrete specimens instrumented with embedded and bonded DOFS cables. Depending on the configuration, these tests allowed us to determine the initial bond properties of the cable/concrete or cable/adhesive interfaces (i.e., the tangential stiffness and the bond strength, see determination method in Section 2.3.1). Experimental values are reported in Table 6.

These results show that tangential stiffness is significantly higher in the bonded cable configuration compared to the embedded one, despite the large dispersion of experimental values. The DOFS cable appears to adhere better in the adhesive than in the concrete. However, the bond strength values are almost similar for both configurations. In all cases, the relief of the cable surface favors mechanical anchoring/interlocking with the surrounding medium and ensures effective bond with both concrete and adhesive.

### 3.2. Characterizations at Different Ageing Times

#### 3.2.1. Mechanical Characterization of Aged Concrete Cylinders

**Figure 12:**
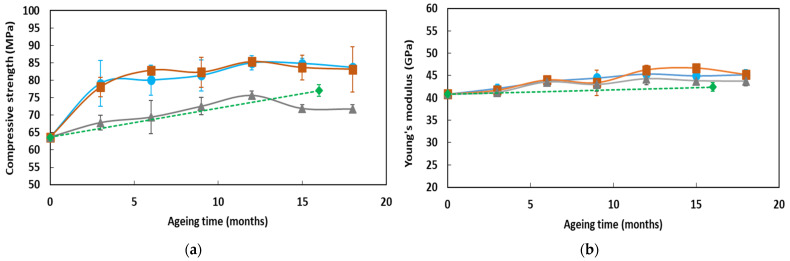

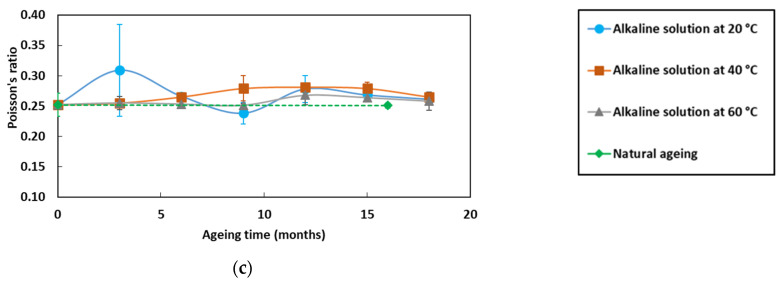
Evolutions of the mechanical characteristics of concrete over exposure in the alkaline and NA environments: (**a**) compressive strength, (**b**) Young’s modulus and (**c**) Poisson’s ratio.

Compression tests were performed on concrete cylinders after several exposure times (T1 to T6) in the alkaline solution at the different temperatures, and after the same exposure periods under outdoor NA. Figure 12 displays the evolutions of the concrete properties (compressive strength, elastic modulus, and Poisson’s ratio) as a function of exposure time. The numerical values are also reported in Table A1 of Appendix A.

These experimental data show the following trends:

- Alkaline environments at 20 and 40 °C favor the hydration process of the cementitious matrix and lead to an increase in compressive strength over time [40], up to a maximum value in the range 80–85 MPa. In the solution at 60 °C, and during NA that generally includes periods with low RH levels, the compressive strength also increases over time, but to a lower extent as the maximum values are in the range 70–75 MPa;
- Changes in the Young’s modulus and Poisson’s ratio during ageing remain limited and seem little dependent on the type of ageing condition. Only a slight increase in Young’s modulus is observed over time, while the Poisson’s ratio does not vary significantly.

#### 3.2.2. Mechanical and Physico-Chemical Characterizations of Aged DOFS Cables

This section investigates the effects of the various ageing environments on the mechanical properties of the component materials and internal interfaces of the DOFS cable. Additional characterizations were also carried out to identify the chemical/physical processes at the molecular scale that are responsible for the observed changes in mechanical characteristics.

**Figure 13:**
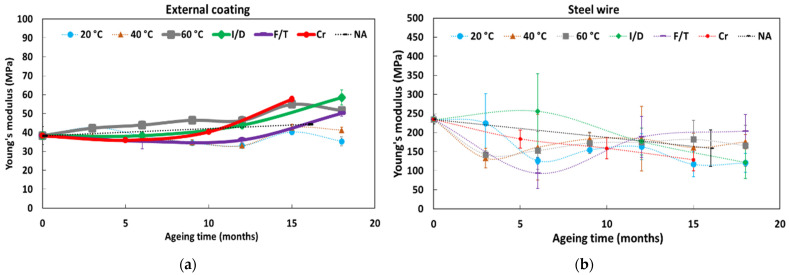

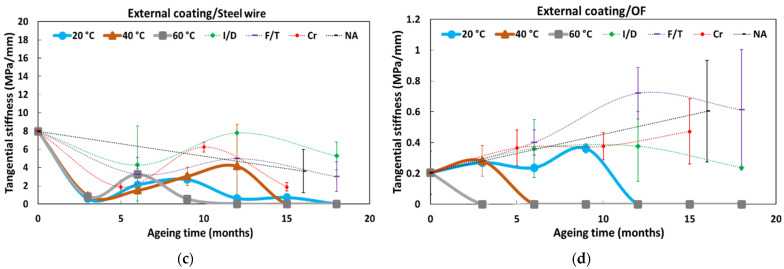
Changes in the mechanical properties of the component materials and internal interfaces of the DOFS cable over exposure in the various ageing environments: tensile Young’s moduli of (**a**) the cable coating and (**b**) the steel wire reinforcement; tangential stiffness of the (**c**) coating/steel wire and (**d**) coating/OF interfaces.

Figure 13 displays the evolutions versus exposure time in the various environments of the mechanical characteristics of the cable’s component materials (Young’s moduli of both the external coating and the steel wire reinforcement, as determined from tensile tests) and of the characteristics of the internal interfaces as well (tangential stiffness of the external coating/steel wire and external coating/OF interfaces, as determined from the linear part of the pull-out test curves (cf. Figure 5b)). To facilitate reading, curves showing major evolutions are represented in plain lines, whereas curves showing little evolution are displayed in dotted lines. 

The numerical values are also reported in Table A1 of Appendix A.

From these experimental results, the following trends can be pointed out:

- The Young’s modulus of the external coating is found to increase during ageing in the alkaline solution at 60 °C, under Cr, and under I/D cycles and F/T cycles, with a more pronounced trend after 8–10 months of exposure. Meanwhile, the value of the Young’s modulus remains almost constant when the cable is exposed to the alkaline solutions at 20 and at 40 °C, or under NA;
- As expected, no significant change in the tensile Young’s modulus of the steel wire reinforcement is observed after ageing, whatever the type of environment;
- Regarding the tangential stiffness of the external coating/steel wire interface, a rapid decrease is observed in the case of DOFS cables immersed in the alkaline solution (the same trend is obtained at all temperatures), leading to zero stiffness at test session T1 (i.e., after only 3 months of exposure). Then, surprisingly, a partial stiffness recovery is observed at session T2. The stiffness then drops again to zero at session T3 for specimens conditioned at 60 °C, while it continues to recover until sessions T3 and T4 in the case of immersions at 20 and 40 °C, respectively. At the end, all immersion conditions lead to a complete and permanent loss of the interface properties (i.e., zero stiffness). It is worth noting that the damage kinetics under immersion seem faster at higher temperature, especially at 60 °C, suggesting that temperature acts as an accelerating factor of the interfacial degradation process. This may result from the penetration of the alkaline solution at the ends of the immersed DOFS cable, and from the subsequent infiltration of this solution by capillary diffusion along the internal interfaces of the DOFS cable. Regarding the other ageing conditions (i.e., F/T and I/D cycling, NA), only a slight decrease in tangential stiffness is observed over exposure time, which may result from moisture diffusion (I/D), from differential thermal expansion of materials involved in the interface behaviour (F/T), or from the coupling of these two processes (NA). In addition, the differential creep behaviour between the external coating and the steel wire reinforcement can also induce differential displacement at the external coating/steel wire interface and may explain the loss of tangential stiffness induced by Cr ageing;
- Regarding the external coating/OF interface, a loss of tangential stiffness is also observed in the case of DOFS cables conditioned in the alkaline solution, with a temperature dependent damage kinetics. Here, again, interfacial degradation can be attributed to the infiltration of the solution into the cable from the cut ends, according to a temperature dependent diffusion kinetics. This process may be accentuated by the pre-existing gaps that were detected by optical microscopy between the OF and the external coating (cf. Figure 1b). Meanwhile, in the other ageing conditions, the tangential stiffness of the external coating/OF interface appears to increase over time, but this trend remains difficult to assert due to the large dispersion on experimental data.

**Figure 14:**
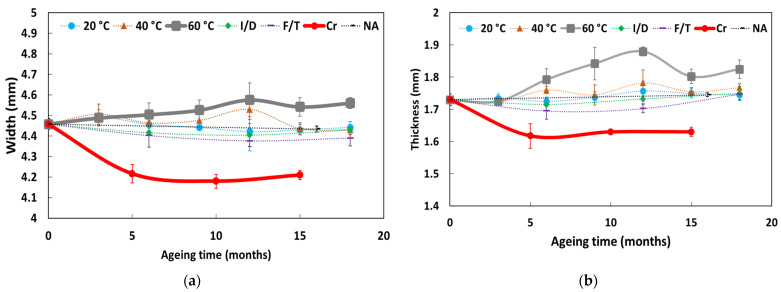
Dimensional changes in the DOFS cable cross-section versus exposure time in the various ageing environments: (**a**) width and (**b**) thickness.

In addition, Figure 14 depicts the dimensional changes in the DOFS cable cross-section as a function of exposure time in the various ageing environments. A relative dimensional stability can be observed after ageing, with the exception of the cables immersed in the alkaline solution at 60 °C or subjected to Cr. In the former case, a progressive swelling of the cable is indeed observed, the cause of which will be investigated later by complementary physico-chemical analyses. For samples subjected to Cr, the cross-section of the cable decreases significantly during the first 5 months and then stabilizes; this phenomenon can be attributed to a plastic elongation of the flexible coating of the cable by Poisson’s effect, under permanent tensile load.

**Figure 15:**
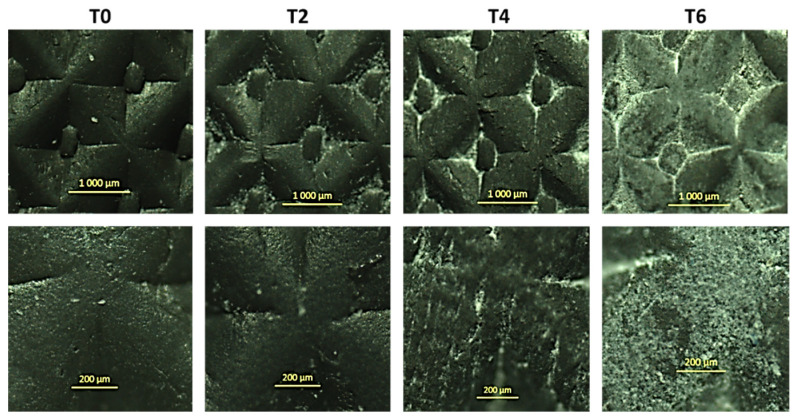
Optical micrographs at different magnifications, of the surface of the DOFS cable in the initial state (T0) and after 6 months (T2), 12 months (T4) and 18 months (T6) of exposure in the alkaline solution at 60 °C.

Observations of the external coating of DOFS cables by optical microscopy did not reveal any significant change in the surface aspect after ageing, except for samples conditioned in the alkaline solution at 60 °C. Figure 15 shows the external surface of the DOFS cable after different exposure periods in this peculiar condition.

Before ageing, the external cable surface appears to be relatively smooth and shiny, and mainly shows the embossment intended to maximize the bond with concrete. Regarding samples aged in the alkaline solutions at 60 °C, micro-scratches appear after 6 months of exposure and their number increases afterwards, conferring to the surface of the coating a granular and matt aspect. In addition, these micro-scratches become filled with grains of crystallized salts coming from the alkaline solution and which strongly adhere to the surface of the cable.

Additional tests were performed to investigate the chemical or microstructural mechanisms involved in the ageing process of the cable coating, especially under immersion in the alkaline solution at 60 °C. The results are presented in the following.

**Figure 16:**
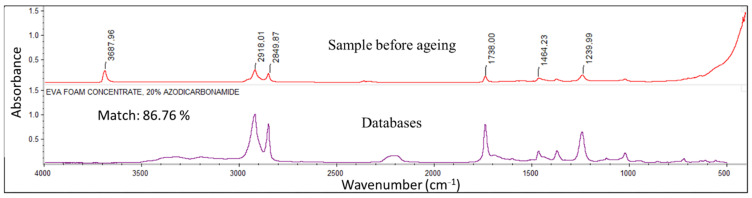
FTIR-ATR spectrum obtained for the external coating of the unaged DOFS cable (at session T0), together with the spectrum from FTIR databases providing the best match.

Figure 16 shows a typical FTIR-ATR spectrum of the surface of the unaged DOFS cable, as well as the spectrum identified from available FTIR databases that provides the best correspondence.

The FTIR spectrum of the cable coating shows several absorption bands that are characteristic of chemical groups present in ethylene vinyl acetate (EVA) copolymers. In particular, one can identify two peaks around 2850 and 2920 cm^−1^ corresponding to the stretching vibrations of methylene -CH_2_- groups, a band at 1738 cm^−1^ corresponding to the stretching of C=O carbonyls present on the acetate groups, and a band around 1240 cm^−1^ relating to the stretching vibration of C–O from vinyl acetate groups. In addition, a good correspondence is obtained (more than 80% match) between the spectrum of the unaged cable coating and several spectra of EVA type copolymers referenced in the FTIR databases. It is therefore possible to state that the cable’s external coating is mainly composed of an EVA copolymer.

Typically, EVA copolymers contain a between 10% and 40% mass fraction of vinyl acetate, with the rest consisting of ethylene units. EVA copolymers are more elastomeric than polyethylene, because vinyl acetate reduces the crystallinity and therefore the stiffness of the material, thus providing increased flexibility and better impact resistance. Their density is correlated with the vinyl acetate content [41].

**Figure 17:**
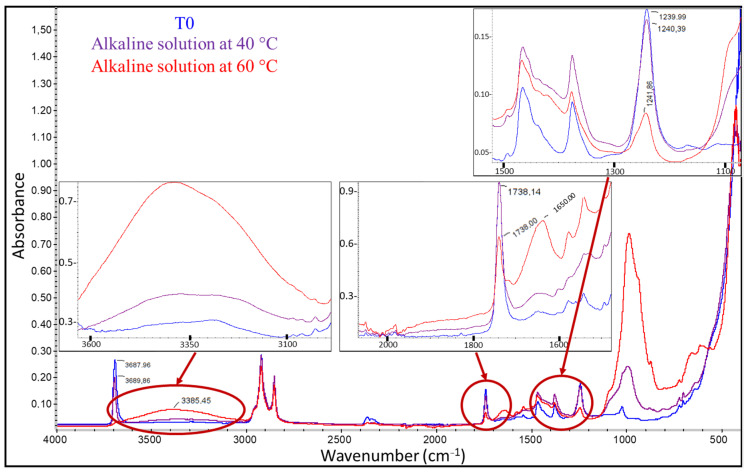
FTIR-ATR spectra obtained for the unaged cable coating (T0) and after 12 months of ageing (T4) in the alkaline solution at 40 and 60 °C.

FTIR analyses were then performed on the external coating of DOFS cables that had been exposed to the various ageing environments. In general, the spectra did not reveal any significant alteration of the chemical structure of the coating after ageing, except for cables immersed in the alkaline solution at 40 and 60 °C. Figure 17 shows the typical FTIR-ATR spectra obtained for the external coating of cables after 12 months exposure (T4) to these two peculiar conditions, as well as the spectrum of the unaged coating for comparison. Zooms corresponding to specific regions of interests of these spectra are also displayed in this figure.

The spectra of samples aged in the alkaline solution at 60 °C show a clear decrease in the intensity of the absorption bands at 1738 and 1240 cm^−1^ relative to the ester groups (vinyl acetates), as well as an increase in the peak height at 1650 cm^−1^ indicating the formation of carboxylic acids. These effects are also present for samples immersed at 40 °C, but to a lower extent, suggesting that the underlying reactional process is activated by temperature. Vinyl acetate groups, which are esters, are known to be very sensitive to various environmental factors such as temperature, ultraviolet (UV) radiation, or oxygen. They can easily decompose to generate free radicals, which can then form different carboxylic acids [42,43]. In the present case, FTIR analyses confirm that the thermal degradation of ester groups occurred for samples immersed in the alkaline solution at 40 and 60 °C.

**Figure 18:**
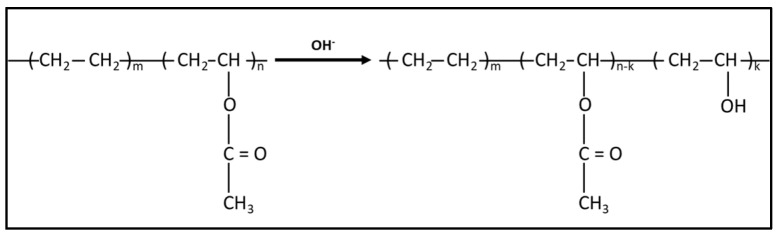
Hydrolysis of EVA copolymers, according to [45].

In addition, the FTIR spectra of the aged samples also show the appearance of a broad band in the region of 3000–3600 cm^−1^ whose amplitude increases with the temperature of the alkaline solution. This band may be ascribed to an additional degradation process by hydrolysis of the acetate groups, leading to the formation of hydroxyl groups according to the mechanism described in Figure 18 [44,45]. This decomposition process of acetates would contribute, similarly to thermal degradation, to decreasing the intensity of the peaks at 1738 and 1250 cm^−1^. Moreover, this hydrolysis phenomenon may also be responsible for the swelling observed for cables aged in the alkaline solution at a high temperature (see Figure 14) [45].

It should be noted that FTIR-ATR enables a characterization of the extreme surface of the analyzed samples, and therefore only the surface of the coating in contact with the alkaline solution are involved in the previous chemical degradation processes. These processes do not necessarily occur (or maybe to a lower extend) in the depth of the cable coating. Such a localized degradation of the coating (mostly restricted to the surfaces in contact with the solution) is consistent with the fact that accelerated ageing in the alkaline solution mainly affects the mechanical properties of internal interfaces (coating/OF or coating/steel wire interfaces, along which the solution may penetrate by capillary diffusion from the ends of the cable), whereas the elastic modulus of the bulk coating material does not deteriorate with ageing (Figure 13). As previously highlighted, the elastic modulus seems even to increase after long exposure periods in the solution at 60 °C, or under cyclic conditions. This phenomenon suggests that some microstructural changes occur in the bulk coating under these conditions. To address this hypothesis, DSC analyses were carried out on small samples taken from the entire section of the cable coating at T0 and after various ageing periods. 

**Figure 19:**
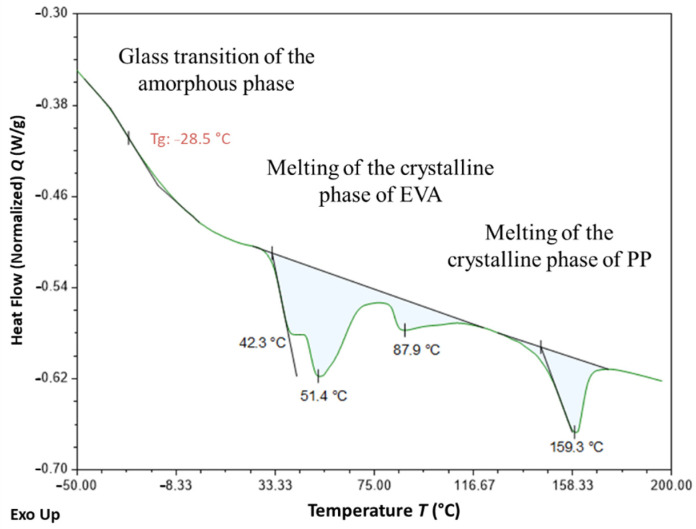
DSC thermogram of the unaged cable coating (T0).

Figure 19 shows the DSC thermogram obtained for the unaged coating (at T0), which highlights several distinctive features:

- An enthalpic jump is visible around −30 °C, which can be attributed to the glass transition of the amorphous phase of the EVA copolymer;
- A large endothermic region is observed between −30 and 110 °C, from which three distinct peaks emerge at approximately 40, 50 and 90 °C. This domain corresponds to the melting temperature range generally reported for the crystalline phase of EVAs [42,43,46]. The presence of multiple peaks reveals the existence of several populations and sizes of crystalline lamellae.
- Another endothermic peak is observed at a higher temperature, around 160 °C, which is not characteristic of the EVA crystal structure. This temperature range is usually associated with the melting of polypropylene (PP), which suggests that the cable coating is actually composed of an EVA/PP copolymer [47].

**Figure 20:**
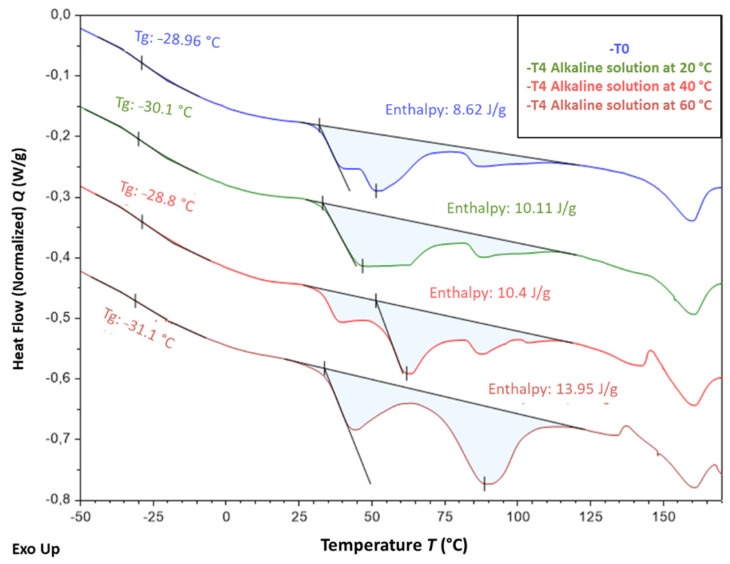
DSC thermograms of the cable coating conditioned for 12 months (T4) in the alkaline solution at different temperatures, and thermogram of the control sample (T0).

Figure 20 shows the typical thermograms of samples that were conditioned for 12 months in the alkaline solution at the three different temperatures. These ageing conditions obviously had a significant impact on the crystal structure of EVA and affected the size distribution of crystalline lamellae. Indeed, the positions of the different melting peaks (in the temperature range from −30 to 110 °C) are significantly influenced by the temperature of the solution. In particular, conditioning at 40 and 60 °C increase the amplitude of melting peaks located around 60 °C and 90 °C, respectively, which can be attributed to the growth of populations of thicker lamellae during ageing. This phenomenon was also accompanied by an increase in the overall degree of crystallinity $\chi_{c}$ of the EVA copolymer, from 4.1% in the unaged state up to 4.6% and 6.0% for specimens immersed at 40 and 60 °C, respectively ($\chi_{c}$ was determined according to Equation (1) [46], where $\Delta H_{m}$ is the total melting enthalpy determined from DSC thermograms and $\Delta H_{m}^{*}$ is the melting enthalpy of a perfect polyethylene crystal, i.e., 277.1 J/g)

(1) $$\chi_{c}=\frac{\Delta H_{m}}{\Delta H_{m}^{*}}$$

Such alterations of the crystal structure may result from recrystallization processes [46], probably activated by temperature, but also from the degradations at molecular scale previously identified by FTIR (chain scissions induced by thermo-degradation of acetate groups and by hydrolysis process). 

It is well known that the mechanical properties of semi-crystalline polymers are mainly controlled by the degree of crystallinity of the material, since crystalline lamellae play a reinforcing role for the amorphous phase. Based on previous DSC results, one can thus assume that the increase in tensile Young’s modulus observed after ageing for the bulk coating material (cf. Figure 13) results from an increase in the overall crystallinity degree of the EVA copolymer due to recrystallization processes.

**Figure 21:**
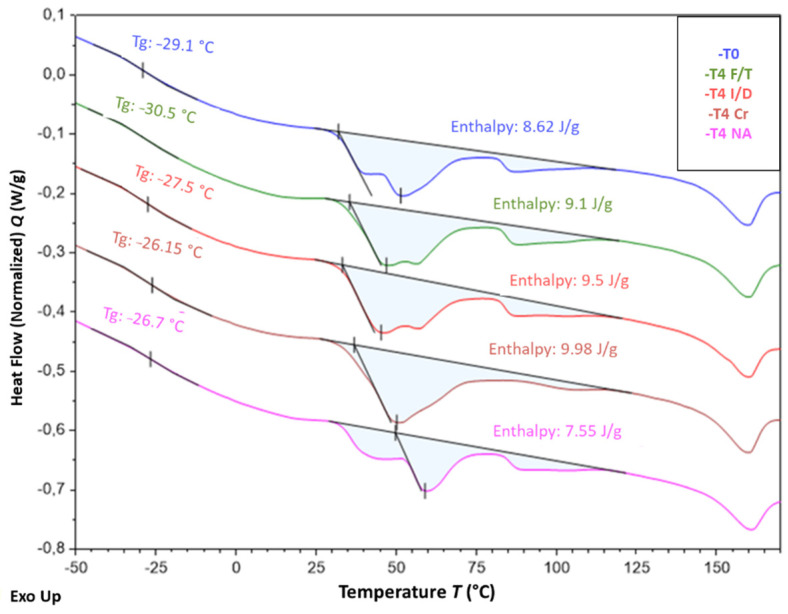
DSC thermograms of the cable coating after 12 months exposure (T4) to F/T, I/D, Cr and NA conditions. The control sample (T0) is also displayed for comparison.

Figure 21 presents the DSC thermograms obtained for samples that were exposed for 12 months to the other ageing environment, i.e., F/T and I/D cycling, as well as Cr and NA conditions. As before, alterations of the melting peaks of aged specimens can be detected on the thermograms, attesting for changes in the lamellae size distribution associated with the crystalline structure of EVA. However, these changes seem less important than those observed after ageing in the alkaline solution at a high temperature. In the case of NA condition, such transformations of the crystal structure are generally activated by photo-oxidation [46].

#### 3.2.3. Characterization of Aged Adhesive Samples

**Figure 22:**
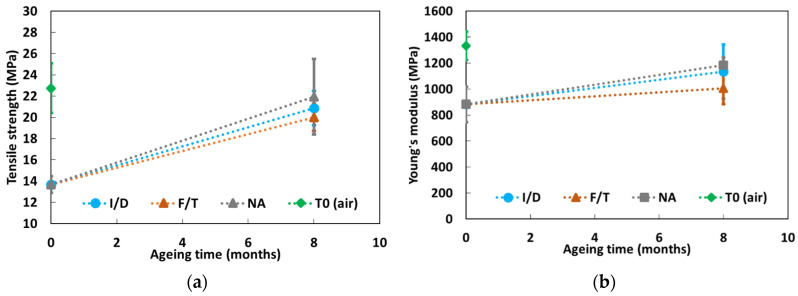
Values of (**a**) the tensile strength and (**b**) Young’s modulus of the X120 adhesive before and after exposure to I/D, F/T and NA conditions for a period of 8 months (T3). The initial state corresponds to preliminary conditioning in water (or in air) for 3 months.

Tensile tests were carried out on dumbbell specimens of the bulk X120 adhesive, both in the initial state (T0) and after 8 months exposure (T3) to the same conditions as instrumented concrete prisms (i.e., F/T and I/D cycles, as well as NA). The objective was to assess possible evolutions of the mechanical properties of the adhesive (tensile strength and Young’s modulus) during ageing. The test results are presented in Figure 22. It should be remembered that the initial state prior to aging (T0) corresponds to a preliminary conditioning of the adhesive samples in water for 3 months, aiming to reproduce the actual curing schedule of concrete prisms with bonded DOFS cables. Additional tests were also performed at T0 on adhesive samples that were previously conditioned in air for three months (i.e., in laboratory conditions at 20 °C and ~50% RH). Corresponding values are denoted T0 (air) in Figure 22 and are provided for the sake of comparison only. 

As previously discussed in Section 3.1.3, the preliminary conditioning of samples in water negatively affected the mechanical properties of the epoxy adhesive at T0, compared to the specimens that were conditioned in air. This effect can mainly be attributed to the plasticization of the polymer network by water molecules, as a result of the high water absorption of the material (~8.5% mass uptake after 3 months in water).

During ageing, hygric conditions were much less severe than in the case of a permanent immersion: indeed, the maximum RH level was about 70% in the framework of F/T cycles, there was a one week drying period every two weeks for the I/D condition, and there was also an alternation of dry/moist periods during NA. In this context, aged specimens showed substantial gains in mechanical properties (Young’s modulus and tensile strength) compared to control specimens at T0, due to partial desorption during ageing, but without fully recovering the values of dry samples (denoted T0 (air) in Figure 22). In addition, exposure to NA conditions seems to promote higher recovery of mechanical properties than exposure to I/D and F/T cyclic conditions, despite the large dispersion on experimental data shown in Figure 22.

Furthermore, complementary physicochemical characterizations were again performed on control and aged adhesive samples to assess the possible chemical degradations and microstructural changes taking place during ageing. 

FTIR analyses did not reveal any significant alteration of the chemical structure of the X120 adhesive subjected to the cyclic an NA conditions. Only slight differences in the intensity of the hydroxyl band between 3000 and 3700 cm^−1^ were detected among the analyzed samples, due to variations of the moisture content.

**Figure 23:**
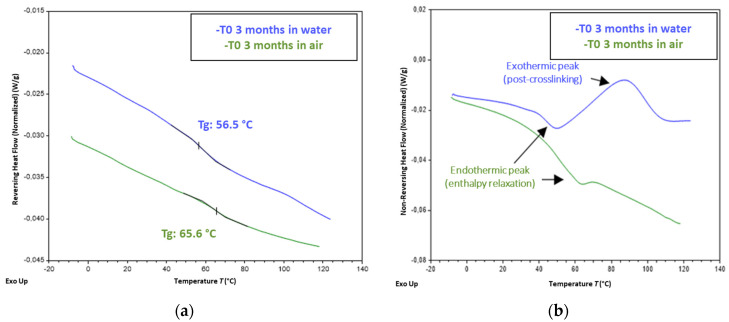
MDSC thermograms obtained for X120 adhesive samples at T0, after preliminary conditioning for 3 months in water or in air: (**a**) reversing and (**b**) non-reversing heat flow signals.

MDSC analyses (see the experimental protocol in Section 2.3.2) were also performed on samples at T0, and on samples aged for 8 months under F/T, I/D, and NA conditions. Figure 23 compares the MDSC thermograms obtained for adhesive samples at T0, after preliminary conditioning in water for 3 months (that reproduces the curing schedule of concrete prisms instrumented with bonded DOFS cables) or after conditioning in water for the same period. The glass transitions were clearly detected as jumps in the reversing heat flow signal (Figure 23a), which enabled the determination of *Tg* values in the range of 56 °C for the adhesive conditioned in water, and 65 °C for the adhesive conditioned in air. In addition, the non-reversing heat flow component (Figure 23b) displayed a large exothermic peak for the adhesive conditioned in water, reflecting post-crosslinking of the polymer during the test, due to the presence of residual monomers that react upon heating. Meanwhile, this phenomenon was not observed for the adhesive conditioned in air. These features suggest that the adhesive conditioned in water is not fully cured at T0, whereas the material stored in air has almost completed its polymerization process. Such differences in both *Tg* and mechanical properties at T0 as a function of the preliminary conditioning treatment of the adhesive samples (in water or in air) may therefore result from the plasticization of the epoxy network by the absorbed water, as already mentioned, but also from differences in the polymerization kinetics.

**Figure 24:**
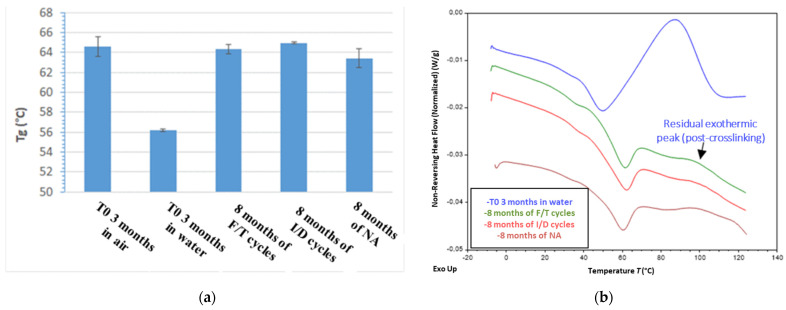
MDSC analyses of X120 adhesive samples exposed for 8 months to F/T, I/D and NA conditions: (**a**) *Tg* values and (**b**) non-reversing Heat flow thermograms.

MDSC analyses were also performed on samples exposed for 8 months to F/T, I/D and NA conditions, which made it possible to determine the *Tg* values of the aged X120 adhesive (Figure 24a). It can be seen that these values (of the order of 65 °C) are significantly higher than *Tg* of the unaged control adhesive at T0 (sample conditioned for 3 months in water) and are close to *Tg* of the dry adhesive at T0 (sample conditioned in air). In addition, the non-reversing heat flow components of the MDSC thermograms are displayed in Figure 24b, and show the presence of a small exothermic peak, accounting for the reaction of residual monomers over heating (post curing phenomenon). However, the enthalpy of this phenomenon (surface area under the peak) is much lower than that obtained for the unaged control adhesive at T0 (sample conditioned in water—see blue curve in Figure 24b). 

From the previous results, one can draw the following conclusions:

- Exposures to F/T, I/D and NA conditions led to the desorption of a large part of the water that was initially present at T0 in the control X120 adhesive (previously conditioned in water),
- Such exposures also favored the cross-linking process of the adhesive during the ageing period, but without reaching complete polymerization of the epoxy network. This phenomenon also explains the observed increase in mechanical properties of aged adhesive samples compared to the unaged control sample at T0 (conditioned in water), and the fact that these properties remain slightly lower than those of the dry adhesive at T0 (sample conditioned in air).

#### 3.2.4. Effect of Ageing on the Bond Properties of DOFS Cable/Concrete and DOFS Cable/Adhesive Interfaces

This section investigates the environmental ageing effects on the bond properties of embedded cable/concrete or bonded cable/adhesive interfaces. Discussions are based on the results of pull-out tests performed on instrumented concrete specimens that were exposed to the various accelerated or NA conditions for periods up to 18 months. At each test session (T1 to T6), three pull-out tests were systematically performed for a given type of aged specimen (i.e., same instrumentation configuration and ageing condition), as summarized in the test program of Table 2. All numerical values are reported in Table A1 of Appendix A.

Overall, the results of these pull-out tests show large experimental dispersion, which makes their interpretation rather difficult. Therefore, the present analysis is mainly focused on some major trends regarding the evolution of the bond properties. These trends are discussed in the light of the changes in physical and mechanical properties of the component materials (concrete, DOFS cable and X120 adhesive) that were previously highlighted.

**Figure 25:**
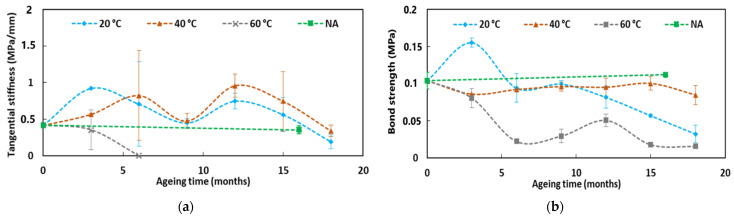
Evolutions of the coating/concrete bond properties during ageing in the alkaline solution at different temperatures or under NA condition: (**a**) tangential stiffness and (**b**) bond strength.

Figure 25 shows the evolution of the bond properties (tangential stiffness and bond strength) at the embedded DOFS cable coating/concrete interface, as a function of the exposure time of pull-out specimens in the alkaline solution at 20, 40 and 60 °C or in the outdoor environment (NA). The graphs make it possible to identify the following trends:

- Regarding specimens immersed in alkaline solution at 60 °C, a severe deterioration of the interfacial properties is observed over the first 6 months of exposure, which can be attributed to surface damage of the cable coating in contact with the interstitial solution of concrete (hydrolysis and thermal degradation of the acetate groups, as revealed by FTIR-ATR). It should be noted that it was not possible to properly determine the tangential stiffness of the coating/concrete interface beyond 6 months of exposure in the alkaline solution at 60 °C. Indeed, at this stage, a very significant degradation of the cable coating/OF interface was noticed due to the infiltration of the alkaline solution from both ends of the DOFS cable (see Figure 13). Consequently, the relative slip of the OF in its coating does not permit us to obtain representative strain measurements (recorded using the OBR interrogation device during the pull-out tests) that are needed for the calculation of *u_t_* (cf. protocol of pull-out tests in Section 2.3.1). Meanwhile, the evolution of the bond strength could be assessed until the last test session T6 (since this value is related to the applied load), and showed a progressive decrease over the course of ageing, without reaching a total degradation/decohesion of the coating/concrete interface;
- For the specimens exposed to the alkaline solution at 20 and 40 °C, only minor changes in the bond properties of the coating/concrete interface were observed for exposure periods up to 15 months. Nevertheless, characterizations performed at the last test session (at T6, after 18 months of exposure) seem also to reveal a significant decreasing trend for both the tangential stiffness and the bond strength;
- Under NA conditions and considering the large dispersion of experimental data, no significant evolution of the coating/concrete bond properties was observed after 16 months of exposure. This result shows that NA, which reproduces actual service conditions in an outdoor environment, does not constitute an aggressive environment for the embedded DOFS cable. In addition, it suggests that immersion in the alkaline solution at 60 °C is a very harsh condition which is not representative of the actual service environment of an embedded FO instrumentation. Nevertheless, additional experiments involving long-term exposure to NA conditions would be necessary to support this conclusion.

**Figure 26:**
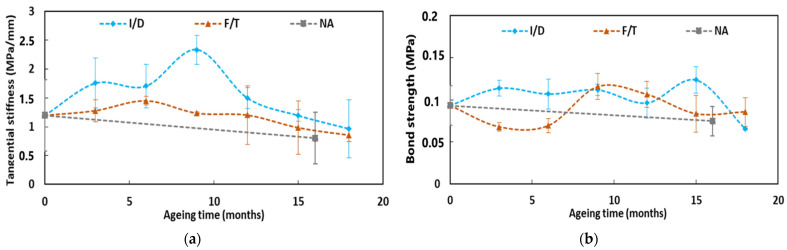
Evolutions of the coating/adhesive bond properties during ageing under I/D, F/T and NA exposure conditions: (**a**) tangential stiffness and (**b**) bond strength.

In the same way, Figure 26 presents the evolution of the bond properties (tangential stiffness and bond strength) at the cable coating/adhesive interface, as a function of the exposure time of pull-out specimens in the I/D, F/T, and NA environments.

As a first result, a progressive enhancement of the tangential stiffness can be noticed over the first 9 months of exposure to I/D cycling conditions. However, beyond 9 months, the peak value gradually decreases. 

On the whole, given the large dispersion of experimental results and comparing values obtained in the initial state (T0) and at the last test session (T6, corresponding to 16 months of exposure for NA and 18 months for I/D and F/T), the graphs show limited changes in the coating/adhesive bond properties after ageing, whatever the exposure condition. Nevertheless, slight decreases in both tangential stiffness and bond strength can be noticed in the last stages for all ageing conditions. Several factors could explain these slight decreases: the effects of water and thermal cycles in the case of I/D and F/T cycles, and the additional effect of UV radiation in the case of NA condition. 

These slight decreases do not seem to be correlated with the changes in the mechanical properties of the X120 adhesive during ageing, which show the opposite trend. Indeed, it was previously reported that the Young’s modulus of the adhesive increases with ageing due to desorption of the specimens and the continuation of the cross-linking process.

### 3.3. Impact of Environmental Ageing on the Strain Response of the DOFS Instrumentation

This last part of the paper illustrates how the ageing-induced degradations/alterations that have previously been evidenced can affect the strain response of the DOFS instrumentation embedded in/bonded to a host concrete structure.

**Figure 27:**
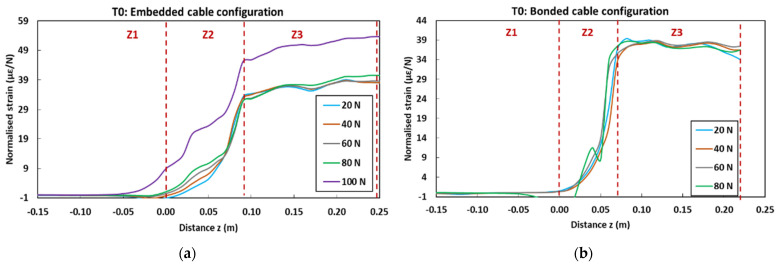
Strain profiles recorded at T0 (initial state before ageing) during the pull-out tests on control concrete specimens instrumented with (**a**) embedded and (**b**) bonded DOFS cables.

Figure 27 displays the strain profiles recorded at T0 (initial state before ageing) during the pull-out tests on control concrete specimens instrumented with embedded and bonded DOFS cables. In the graphs, each curve corresponds to the strain profile recorded by the OBR device at a selected load level (strain is normalized by the considered load value). The curves show three distinct zones (Z1, Z2 and Z3) corresponding to specific domains along the *z*-axis of the specimens (cf. geometry of the specimens in Figure 6):

- Z1 represents the part of the cable not in contact with concrete, and hence is not subjected to any mechanical stress nor strain;
- Z2 represents the segment of the cable in contact with the host concrete (i.e., contact lengths of 9 cm for the embedded cable configuration and 7 cm for the bonded cable configuration). A continuous strain evolution is observed in this zone, which accounts for the shear load transfer process between the core OF and the host concrete structure through the intermediate layers of the DOFS cable, and through the adhesive as well in the case of the bonded configuration.
- Finally, Z3 corresponds to the part of the cable subjected to uniform tensile stress, the end of which is clamped by the upper grip of the UTM. Both stress and strain levels are roughly constant in this zone.

Overall, the strain profiles recorded at various load levels become superimposed once normalized. Nevertheless, the application of high load levels induces significant damage to the system, and hence alters the normalized strain profiles. For instance, at 100 N for the embedded cable configuration (Figure 27a), a loss of adhesion occurs at the coating/OF interface, leading to the enlargement of zone Z2; in addition, a large increase in the normalized strain value is observed in zone Z3, due to substantial decohesion at the coating/steel wire interface.

**Figure 28:**
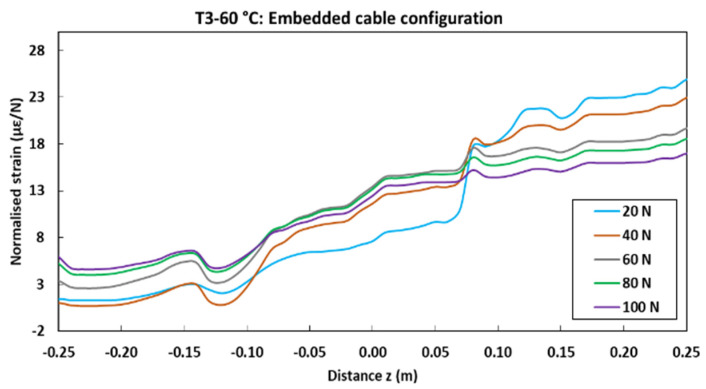
Strain profiles recorded during the pull-out tests on concrete specimens instrumented with embedded DOFS cables after 9 months of immersion (T3) in the alkaline solution at 60 °C.

Figure 28 displays the normalized strain profiles recorded during pull-out tests performed on concrete specimens with embedded DOFS cables after 9 months of immersion in the alkaline solution at 60 °C. A comparison of this graph with the initial strain profile (Figure 27a) clearly demonstrates the impact of these harsh ageing conditions on the strain transfer process between the OF and the host concrete. Indeed, it becomes difficult to identify the different zones (Z1, Z1 and Z3) along the measurement line after ageing, as the load transfer becomes almost uniform over the entire cable length. This phenomenon can be mainly attributed to the slip of the OF within the cable coating, resulting from the degradation of the bond properties at internal interfaces caused by the penetration of the alkaline solution into the cable (cf. Section 3.2.2). The loss of bond properties at the concrete/coating interface that was observed beyond 6 months of exposure in this specific condition (cf. Section 3.2.4) may have also contributed to the observed alteration of the strain profiles. 

**Figure 29:**
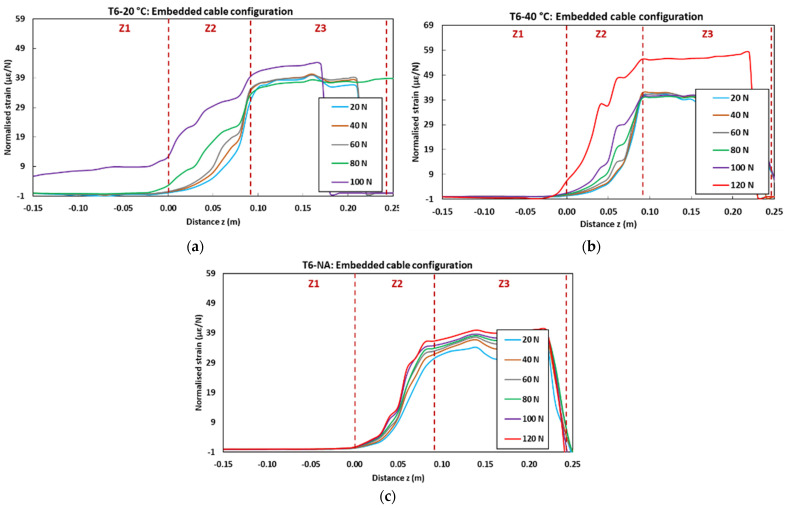
Normalized strain profiles obtained at session T6 during pull-out tests on aged concrete specimens instrumented with embedded DOFS cables: after 18 months immersion in the alkaline solution at (**a**) 20 °C and (**b**) 40 °C, and (**c**) after 16 months of exposure to NA condition.

Figure 29 presents the normalized strain profiles recorded during pull-out tests at the last session, T6 (i.e., after 18 months of exposure to accelerated ageing conditions, or after 16 months in the NA environment), for aged specimens with embedded DOFS cables.

It is worth noting that DOFS cables could not be interrogated for specimens aged in the alkaline solution at 60° C due to an extensive degradation, and hence no graph is presented for this peculiar case.

For specimens aged in the solution at 20 and 40 °C, comparison with profiles of the unaged control specimen (Figure 27a) shows a more limited impact of the long-term exposure. One can observe a slight enlargement of zone Z2, and the fact that normalized curves do not coincide any more in this zone (the normalized strain level seems to increase with the applied load). These features are consistent with the substantial degradation of the interfacial bond properties within the cable, as shown in Section 3.2.2.

Profiles obtained for specimens aged in the NA environment do not reveal significant alteration compared to those of the control specimen, confirming the previous statement that NA is not a severe condition for the embedded DOFS instrumentation.

**Figure 30:**
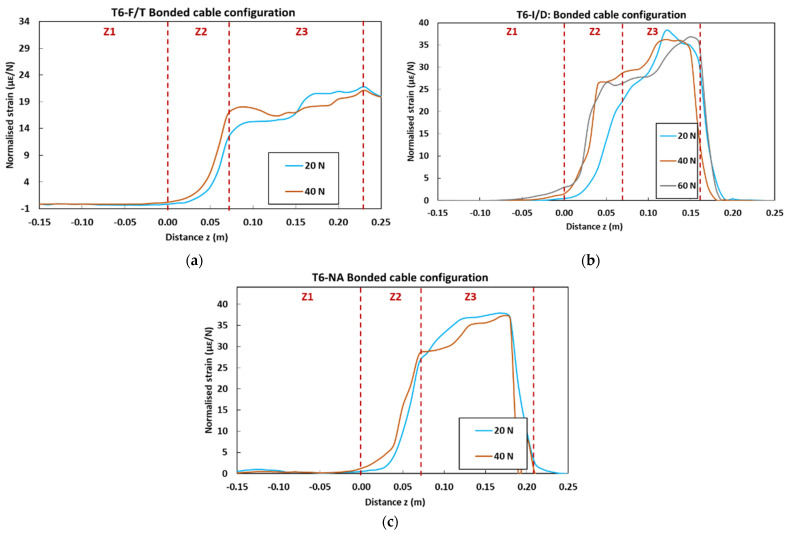
Normalized strain profiles obtained at session T6 during pull-out tests on aged concrete specimens instrumented with bonded DOFS cables: after 18 months of exposure to (**a**) F/T and (**b**) I/D cycles, and (**c**) after 16 months of exposure to NA condition.

In the same way, Figure 30 displays the normalized profiles obtained at T6 for aged pull-out specimens instrumented with bonded DOFS cables.

For specimens exposed to F/T and I/D cycles, and to NA conditions as well, it is found that the number of load levels at which the DOFS instrumentation could be interrogated is reduced in comparison with the unaged control specimen (Figure 27b). This is consistent with an early failure of the cable coating/adhesive interface, in line with the slight decrease in bond properties previously highlighted by the pull-out tests (Figure 26). In addition, the maximum level of the normalized strain in zone Z3 seems also to be significantly reduced compared to the reference, suggesting a less effective strain transfer.

Therefore, the strain response of bonded DOFS instrumentation seems significantly affected by a long-term exposure to external weather conditions, contrary to the embedded configuration.

## 4. Conclusions

This paper presents the results of an extensive experimental program aiming at investigating the environmental durability of a commercial DOFS cable intended for distributed strain measurements in RC structures. Two configurations were considered for the DOFS instrumentation, corresponding to actual applications in the field: DOFS cables embedded in the concrete structure, and cables bonded to the concrete surface. Regarding the embedded configuration, different types of specimens (concrete cylinders, bare DOFS cables, and pullout specimens with embedded cables) were subjected to accelerated ageing in an alkaline solution at different temperatures, in order to simulate the alkaline cement environment. In the same way, for the bonded configuration, specific specimens (samples of bulk adhesive, bare DOFS cables, and pull-out specimens with bonded DOFS cables) were exposed to I/D and F/T cycles to simulate climatic conditions. In addition, a series of previous specimens were subjected to NA in an outdoor environment for the sake of comparison with accelerated exposure tests. Mechanical tests were periodically carried out on these series of specimens (test sessions T0 to T6, with a maximum exposure time of 18 months) to assess the evolution after ageing of the mechanical properties of individual component materials (concrete, component materials and internal interfaces of the DOFS cable, adhesive used to bond the cable), as well as the evolution of the bond properties at the cable coating/concrete and cable coating/adhesive interfaces. The observed evolution of mechanical properties after ageing were then analyzed in the light of chemical degradations/microstructural alterations of the component materials revealed by complementary physico-chemical characterizations. The main conclusions of this durability study are summarized in the following.

Regarding the embedded DOFS configuration, it was found that:

- Accelerated ageing in the alkaline solution affected the bond properties at internal interfaces of the DOFS cable (coating/OF and coating/steel wire interfaces), as well as the bond properties between concrete and the embedded cable, with deterioration kinetics that followed temperature dependent trends. For instance, the bond between the coating and the core OF became almost negligible after only 3 months of exposure in the solution at 60 °C, while similar deterioration was observed after 6 months at the concrete/coating interface. FTIR analyses showed that the component material of the coating is an EVA copolymer, which undergoes chemical degradations by hydrolysis and thermal degradation of acetate groups. These degradations seem restricted to the surfaces of the coating in direct contact with the alkaline solution, i.e., at the internal interfaces of the cable (due to the infiltration of the solution by capillary diffusion from the ends of the cable, favored by the preexisting gaps between the coating and the OF), or at the cable/concrete interface where the coating is in contact with the interstitial pore solution. Such surface degradations explain the deterioration of interfacial bond properties, and inevitably modify the strain transfer process between the host structure and the core OF.
- Conversely, the mechanical properties of the bulk coating material itself were little affected after ageing in the alkaline solution, and even increased after long-term exposure, which was attributed to a temperature activated alteration of the crystalline structure and an overall increase in the crystallinity degree of the EVA copolymer, as evidenced by DSC.
- Unlike accelerated conditions, the exposure of the DOFS cable and pull-out specimens with embedded cables to the NA environment for periods up to 16 months did not show clear evolutions of the bond properties, either at the internal interfaces of the cable nor at the coating/concrete interface. This result suggests that direct immersion in the alkaline solution, especially at elevated temperatures, is a very harsh environment which may lead to an overestimation of environmental effects compared to actual service conditions.
- Regarding the bonded configuration DOFS, results showed the following trends:
- Accelerated ageing under F/T and I/D cycling for periods up to 18 months only little affected the bond properties at the internal interfaces of the DOFS cable, or even induced a slight increase which may not be very significant due to the large dispersion of experimental data. The bond properties between the cable and the epoxy adhesive also remained around their initial level after 18 months of exposure. In addition, the mechanical properties of the bulk adhesive increased with ageing, compared to the initial state at T0, due to sample desorption and to the post-cure of the polymer network.
- Meanwhile, NA conditions seemed to slightly deteriorate the bond properties between the cable and the adhesive. This different behavior may result from coupling effects of temperature and humidity and from the action of UV radiations, which took place in the outdoor environment but were not included in the F/T and I/D protocols. Therefore, in future research work, cyclic ageing protocols could be improved by considering these additional environmental factors.

It was also shown that DOFS cables subjected to creep load experienced a significant degradation of the bond properties at the coating/steel wire interface. This may result from the large creep strain of the EVA coating (evidenced by a large reduction in the cable diameter), which induces important differential displacement and possibly decohesion at the cable/steel wire interface. 

Finally, an analysis of the distributed strain profiles collected during pull-out tests on instrumented concrete specimens provided a clear illustration of the consequences of ageing processes on the strain response of the DOFS cable:

- It was shown that immersion in the alkaline solution, especially at elevated temperature, strongly modifies the strain profiles along the embedded DOFS instrumentation, due to deterioration of bond properties at internal interfaces and at the concrete/coating interface as well. Meanwhile, exposure to NA conditions had little effect on recorded strain profiles, confirming that outdoor exposure is not an issue for the durability of the embedded instrumentation.
- Regarding the bonded instrumentation, exposure to accelerated conditions (F/T and I/D cycles) and to NA environment both induced early pull-out failure of the system and seemed to slightly reduce the strain transfer effectiveness.

As a last remark, we would like to remind the reader that the results reported in this article are specific to the peculiar DOFS cable considered in the experimental program. However, the proposed methodology can be reproduced for any commercial cable on the market in order to investigate its specific ageing behavior.

## Figures and Tables

**Table 1:** Chemical composition of the alkaline solution.

Chemical Compound	Concentration
NaOH	0.1 mol/L (4 g/L)
KOH	0.5 mol/L (28.05 g/L)

**Table 2:** Summary of the durability test campaign, showing the characterizations performed at each test session (with the number of repeated tests in brackets).

				Test Sessions						
				T0	T1	T2	T3	T4	T5	T6
Ageing environments	Alkaline solution	*Exposure time (months)*		***0***	***3***	***6***	***9***	***12***	***15***	***18***
		**Tests**	**Concrete: compression tests (x3)**	**X**	**X**	**X**	**X**	**X**	**X**	**X**
			**Embedded DOFS: pull-out tests (x3)**	**X**	**X**	**X**	**X**	**X**	**X**	**X**
			**DOFS cables: tensile tests** **coating (x3) + Steel wire (x3)**	**X**	**X**	**X**	**X**	**X**	**X**	**X**
			**DOFS: pull-out tests** **OF/coating (x3) + Steel/coating (x3)**	**X**	**X**	**X**	**X**	**X**	**X**	**X**
			**Other analyses (OM, DSC, FTIR, D)**	**X**	**X**	**X**	**X**	**X**	**X**	**X**
	F/T cycles	*Exposure time (months)*		***0***	***3***	***6***	***9***	***12***	***15***	***18***
		**Tests**	**Bonded DOFS: pull-out tests (x3)**	**X**	**X**	**X**	**X**	**X**	**X**	**X**
			**DOFS cables: tensile tests** **coating (x3) + Steel wire (x3)**	**X**		**X**		**X**		**X**
			**DOFS cables: pull-out tests** **OF/coating (x3) + Steel/coating (x3)**	**X**		**X**		**X**		**X**
			**Other analyses (OM, DSC, FTIR, D)**	**X**		**X**		**X**		**X**
	I/D cycles	*Exposure time (months)*		***0***	***3***	***6***	***9***	***12***	***15***	***18***
		**Tests**	**Bonded DOFS: pull-out tests (x3)**	**X**	**X**	**X**	**X**	**X**	**X**	**X**
			**DOFS cables: tensile tests** **coating (x3) + Steel rod (x3)**	**X**		**X**		**X**		**X**
			**DOFS cables: pull-out tests** **OF/coating (x3) + Steel/coating (x3)**	**X**		**X**		**X**		**X**
			**Other analyses (OM, DSC, FTIR, D)**	**X**		**X**		**X**		**X**
	Natural aging (NA)	*Exposure time (months)*		***0***				***12***		***16***
		**Tests**	**Concrete: compression tests (x3)**	**X**						**X**
			**Embedded DOFS: pull-out tests (x3)**	**X**						**X**
			**Bonded DOFS: pull-out tests (x3)**	**X**						**X**
			**DOFS cables: tensile tests** **coating (x3) + Steel wire (x3)**	**X**						**X**
			**DOFS cables: pull-out tests** **OF/coating (x3) + Steel/coating (x3)**	**X**						**X**
			**Other analyses (OM, DSC, FTIR, D)**	**X**				**X**		**X**
	F/T, I/D, NA	*Exposure time (months)*		***0***			***8***			
		**Tests**	**Bulk X120 adhesive: tensile tests** **(x3 per aging environment)**	**X**			**X**			
			**Other analyses on X120 (DSC, FTIR)**	**X**			**X**			
	Creep (Cr)	*Exposure time (months)*		***0***		***5***		***10***	***15***	
		**Tests**	**DOFS cables: tensile tests** **coating (x3) + Steel wire (x3)**	**X**		**X**		**X**	**X**	
			**DOFS cables: pull-out tests** **OF/coating (x3) + Steel/coating (x3)**	**X**		**X**		**X**	**X**	
			**Other analyses** **(D, DSC)**	**X**		**X**		**X**	**X**	

**Table 3:** Mechanical properties of control concrete cylinders (diameter 11 cm, height 22 cm).

	Average	Standard Deviation
Compressive strength (MPa)	63.7	0.9
Young’s modulus (GPa)	40.8	0.2
Poisson’s ratio	0.25	0.02

**Table 4:** Initial mechanical characteristics of the material components and internal interfaces of the DOFS cable, obtained from tensile and pull-out tests.

	Average	Standard Deviation
Young’s modulus of the external coating (MPa)	38	5
Young’s modulus of the steel wire reinforcement (GPa)	234	12
Tangential stiffness of the external coating/OF (MPa/mm)	0.21	0.14
Tangential stiffness of the external coating/steel wire (MPa/mm)	7.97	0.11
Bond strength of the external coating/OF (MPa)	0.055	0.004
Bond strength of the external coating/steel wire (MPa)	0.39	0.04

**Table 5:** Tensile properties of the bulk X120 adhesive after conditioning for 3 months in water or in air at 20 °C.

	Specimens Kept in Water		Specimens Kept in Ambient Air	
	Average	Standard Deviation	Average	Standard Deviation
Young’s modulus (MPa)	882	138	1333	109
Tensile strength (MPa)	13.7	0.8	22.7	2.3
Ultimate strain (%)	6.6	2	1.8	0.03

**Table 6:** Initial bond properties at the concrete/cable or adhesive/cable interfaces, obtained from pull-out tests on unaged concrete specimens with embedded or bonded DOFS cables.

	Tangential Stiffness (MPa/mm)		Bond Strength (MPa)	
	Average	Standard Deviation	Average	Standard Deviation
Embedded configuration	0.42	0.03	0.104	0.011
Bonded configuration	1.20	0.62	0.093	0.023

## Appendix A

**Table A1:** Numerical values of the mechanical properties of the various materials and interfaces, obtained from mechanical tests at sessions T0 to T6. Percentage variations compared to the initial properties at T0 are given in brackets.

			Test Sessions						
			T0	T1	T2	T3	T4	T5	T6
Alkaline solution at 20 °C	*Exposure time (months)*		***0***	***3***	***6***	***9***	***12***	***15***	***18***
	**Measured properties**	**Concrete properties** (**see Figure 12**)							
		Compressive strength of concrete (MPa)	63.67	79.11 (+24%)	80.05 (+26%)	81.42 (+28%)	85.02 (+34%)	84.85 (+33%)	83.72 (+31%)
		Young’s modulus of concrete (GPa)	40.82	42.12 (+3%)	43.69 (+7%)	44.46 (+9%)	45.37 (+11%)	44.96 (+10%)	45.22 (+11%)
		**Bond properties at concrete/cable interface** (**see Figure 25**)							
		Tangential stiffness (MPa/mm)	0.42	0.92 (+121%)	0.71 (+69%)	0.45 (+8%)	0.75 (+79%)	0.56 (+35%)	0.19 (−54%)
		Bond strength (MPa)	0.10	0.16 (+49%)	0.09 (−9%)	0.10 (−4%)	0.08 (−22%)	0.06 (−45%)	0.03 (−69%)
		**Tensile properties of DOFS component materials** (**see Figure 13**)							
		Young’s modulus of the coating (GPa)	38.29	42.67 (+11%)	38.33 (0%)	34.55 (−10%)	33.06 (−14%)	40.21(+5%)	35.33 (−8%)
		Young’s modulus of the Steel wire (GPa)	234.26	224.56 (−4%)	126.44 (−46%)	154.78 (−34%)	162.76 (−31%)	116.86 (−50%)	120.73 (−48%)
		**Bond properties at internal interfaces** (**see Figure 13**)							
		Tangential stiffness at OF/coating interface (MPa/mm)	0.20	0.28 (+37%)	0 (−100%)	0 (−100%)	0 (−100%)	0 (−100%)	0 (−100%)
		Tangential stiffness at steel/coating interface (MPa/mm)	7.97	0.93 (−88%)	1.49 (−81%)	3.04 (−62%)	4.15 (−48%)	0 (−100%)	0 (−100%)
Alkaline solution at 40 °C	*Exposure time (months)*		***0***	***3***	***6***	***9***	***12***	***15***	***18***
	**Measured properties**	**Concrete properties** (**see Figure 12**)							
		Compressive strength of concrete (MPa)	63.67	78.07 (+23%)	82.89 (+30%)	82.31 (+29%)	85.35 (+34%)	83.67 (+31%)	83.17 (+31%)
		Young’s modulus of concrete (GPa)	40.82	41.71 (+2%)	44.01 (+8%)	43.39 (+6%)	46.26 (+13%)	46.67 (+14%)	45.22 (+11%)
		**Bond properties at concrete/cable interface** (**see Figure 25**)							
		Tangential stiffness (MPa/mm)	0.42	0.57 (+36%)	0.82 (+97%)	0.48 (+14%)	0.96 (+130%)	0.75 (+79%)	0.34 (−18%)
		Bond strength (MPa)	0.10	0.09 (−18%)	0.09 (−12%)	0.10 (−8%)	0.09 (−9%)	0.10 (−4%)	0.08 (−19%)
		**Tensile properties of DOFS component materials** (**see Figure 13**)							
		Young’s modulus of the coating (GPa)	38.29	38.25 (0%)	37.45 (−2%)	34.76 (−9%)	33.02 (−14%)	42.54 (+11%)	41.28 (+8%)
		Young’s modulus of the steel wire (GPa)	234.26	132.65 (−43%)	161.58 (−31%)	183.40 (−22%)	183.57 (−22%)	160.76 (−31%)	175.63 (−25%)
		**Bond properties at internal interfaces** (**see Figure 13**)							
		Tangential stiffness at OF/coating interface (MPa/mm)	0.20	0.28 (+37%)	0 (−100%)	0 (−100%)	0 (−100%)	0 (−100%)	0 (−100%)
		Tangential stiffness at steel/coating interface (MPa/mm)	7.97	0.93 (−88%)	1.49 (−81%)	3.04 (−62%)	4.15 (−48%)	0 (−100%)	0 (−100%)
Alkaline solution at 60 °C	*Exposure time (months)*		***0***	***3***	***6***	***9***	***12***	***15***	***18***
	**Measured properties**	**Concrete properties** (**see Figure 12**)							
		Compressive strength of concrete (MPa)	63.67	67.89 (+7%)	69.45 (+9%)	72.6 (+14%)	75.76 (+19%)	71.96 (+13%)	71.84 (+13%)
		Young’s modulus of concrete (GPa)	40.82	41.28 (+1%)	43.56 (+7%)	43 (+5%)	44.28 (+8%)	43.83 (+7%)	43.75 (+7%)
		**Bond properties at concrete/cable interface** (**see Figure 25**)							
		Tangential stiffness (MPa/mm)	0.42	0.35 (−15%)	0 (−99%)	0.12 (−71%)	0.13 (−69%)	0.06 (−86%)	0 (−100%)
		Bond strength (MPa)	0.011	0.013 (+13%)	0 (−100%)	0.009 (−21%)	0.008 (−26%)	0 (−100%)	0.003 (−72%)
		**Tensile properties of DOFS component materials** (**see Figure 13**)							
		Young’s modulus of the coating (GPa)	38.29	42.27 (+10%)	43.91 (+15%)	46.49 (+21%)	46.30 (+21%)	54.89 (+43%)	51.70 (+35%)
		Young’s modulus of the Steel wire (GPa)	234.26	142.15 (−39%)	152.84 (−35%)	172.61 (−26%)	174.81 (−25%)	182.05 (−22%)	165.63 (−29%)
		**Bond properties at internal interfaces** (**see Figure 13**)							
		Tangential stiffness at OF/coating interface (MPa/mm)	0.20	0 (−100%)	0 (−100%)	0 (−100%)	0 (−100%)	0 (−100%)	0 (−100%)
		Tangential stiffness at steel/coating interface (MPa/mm)	7.97	0.80 (−90%)	3.25 (−59%)	0.55 (−93%)	0 (−100%)	0 (−100%)	0.00
F/T cycles	*Exposure time (months)*		***0***	***3***	***6***	***9***	***12***	***15***	***18***
	**Measured properties**	**Bond properties at concrete/adhesive interface** (**see Figure 26**)							
		Tangential stiffness (MPa/mm)	1.20	1.28 (+7%)	1.45 (+21%)	1.24 (+3%)	1.20 (0%)	0.99 (−18%)	0.86 (−29%)
		Bond strength (MPa)	0.09	0.07 (−27%)	0.07 (−26%)	0.12 (+24%)	0.11 (+14%)	0.08 (−11%)	0.09 (−8%)
		**Tensile properties of DOFS component materials** (**see Figure 13**)							
		Young’s modulus of the coating (GPa)	38.29		35.41 (−8%)		36.03 (−6%)		50.16 (+31%)
		Young’s modulus of the Steel wire (GPa)	234.26		93.60 (−60%)		188.51 (−20%)		203.90 (−13%)
		**Bond properties at internal interfaces** (**see Figure 13**)							
		Tangential stiffness at OF/coating interface (MPa/mm)	0.20		0.40 (+96%)		0.72 (+252%)		0.61 (+199%)
		Tangential stiffness at steel/coating interface (MPa/mm)	7.97		3.27 (−59%)		4.99 (−37%)		2.99 (−62%)
I/D cycles	*Exposure time (months)*		***0***	***3***	***6***	***9***	***12***	***15***	***18***
	**Measured properties**	**Bond properties at concrete/adhesive interface** (**see Figure 26**)							
		Tangential stiffness (MPa/mm)	1.20	1.76 (+46%)	1.71 (+42%)	2.34 (+95%)	1.50 (+25%)	1.20 (0%)	0.97 (−19%)
		Bond strength (MPa)	0.09	0.11 (+22%)	0.11 (+15%)	0.11 (+20%)	0.10 (+3%)	0.12 (+33%)	0.07 (−30%)
		**Tensile properties of DOFS component materials** (**see Figure 13**)							
		Young’s modulus of the coating (GPa)	38.29		38.27 (0%)		43.80 (+14%)		58.43 (+53%)
		Young’s modulus of the Steel wire (GPa)	234.26		256.10 (+9%)		176.54 (−25%)		121.47 (−48%)
		**Bond properties at internal interfaces** (**see Figure 13**)							
		Tangential stiffness at OF/coating interface (MPa/mm)	0.20		0.36 (+76%)		0.38 (+84%)		0.24 (+16%)
		Tangential stiffness at steel/coating interface (MPa/mm)	7.97		4.28 (−46%)		7.81 (−2%)		5.28 (−34%)
Natural ageing (NA)	*Exposure time (months)*		***0***						***16***
	**Measured properties**	**Concrete properties** (**see Figure 12**)							
		Compressive strength of concrete (MPa)	63.67						77.08 (+21%)
		Young’s modulus of concrete (GPa)	40.82						42.45 (+4%)
		**Bond properties at concrete/cable interface** (**see Figure 25**)							
		Tangential stiffness (MPa/mm)	0.42						0.35 (−15%)
		Bond strength (MPa)	0.10						0.11 (+8%)
		**Bond properties at concrete/adhesive interface** (**see Figure 26**)							
		Tangential stiffness (MPa/mm)	1.20						0.80 (−33%)
		Bond strength (MPa)	0.09						0.07 (−20%)
		**Tensile properties of DOFS component materials** (**see Figure 13**)							
		Young’s modulus of the coating (GPa)	38.29						44.25 (+16%)
		Young’s modulus of the Steel wire (GPa)	234.26						158.75 (−32%)
		**Bond properties at internal interfaces** (**see Figure 13**)							
		Tangential stiffness at OF/coating interface (MPa/mm)	0.20						0.60 (+195%)
		Tangential stiffness at steel/coating interface (MPa/mm)	7.97						3.60 (−55%)
Creep (Cr)	*Exposure time (months)*		***0***		***5***		***10***	***15***	
	**Measured properties**	**Tensile properties of DOFS component materials** (**see Figure 13**)							
		Young’s modulus of the coating (GPa)	38.29		35.97 (−6%)		40.34 (+5%)	57.46 (+50%)	
		Young’s modulus of the Steel wire (GPa)	234.26		183.21 (−22%)		158.72 (−32%)	128.28 (−45%)	
		**Bond properties at internal interfaces** (**see Figure 13**)							
		Tangential stiffness at OF/coating interface (MPa/mm)	0.20		0.37 (+79%)		0.38 (+84%)	0.47 (+131%)	
		Tangential stiffness at steel/coating interface (MPa/mm)	7.97		1.86 (−77%)		6.26 (−21%)	1.87 (−76%)	
